# A theoretical model for host-controlled regulation of symbiont density

**DOI:** 10.1111/jeb.14246

**Published:** 2023-11-13

**Authors:** Mathilda Whittle, Michael B. Bonsall, Antoine M. G. Barreaux, Fleur Ponton, Sinead English

**Affiliations:** 1 School of Biological Sciences, University of Bristol, Bristol, UK; 2 Department of Biological Sciences, Macquarie University, Sydney, New South Wales, Australia; 3 Department of Biology, University of Oxford, Oxford, UK; 4 St Peter’s College, Oxford, UK; 5 UMR INTERTRYP, CIRAD, Montpellier, France; 6 Animal Health Theme, ICIPE, Nairobi, Kenya

**Keywords:** cost–benefit, energy allocation, fitness, optimization, symbiosis

## Abstract

There is growing empirical evidence that animal hosts actively control the density of their mutualistic symbionts according to their requirements. Such active regulation can be facilitated by compartmentalization of symbionts within host tissues, which confers a high degree of control of the symbiosis to the host. Here, we build a general theoretical framework to predict the underlying ecological drivers and evolutionary consequences of host-controlled endosymbiont density regulation for a mutually obligate association between a host and a compartmentalized, vertically transmitted symbiont. Building on the assumption that the costs and benefits of hosting a symbiont population increase with symbiont density, we use state-dependent dynamic programming to determine an optimal strategy for the host, i.e., that which maximizes host fitness, when regulating the density of symbionts. Simulations of active host-controlled regulation governed by the optimal strategy predict that the density of the symbiont should converge to a constant level during host development, and following perturbation. However, a similar trend also emerges from alternative strategies of symbiont regulation. The strategy which maximizes host fitness also promotes symbiont fitness compared to alternative strategies, suggesting that active host-controlled regulation of symbiont density could be adaptive for the symbiont as well as the host. Adaptation of the framework allowed the dynamics of symbiont density to be predicted for other host-symbiont ecologies, such as for non-essential symbionts, demonstrating the versatility of this modelling approach.

## 1 Introduction

Mutualistic microbes provide a range of benefits to their hosts, for example, supporting defence against pathogens (Brownlie & Johnson, 2009; Haine, 2008), aiding digestion (Gaio Ade et al., 2011; Pais et al., 2008), and supplementing limited host diets through the production of micronutrients (Ankrah & Douglas, 2018; Douglas, 2017). In addition to the benefit provided to the host, there are generally costs in maintaining a symbiont (Engl et al., 2020), and the costs and benefits of harbouring a symbiont population can depend on symbiont density (Chong & Moran, 2016; Chrostek et al., 2013; Cunning & Baker, 2014; Drew & King, 2022; Martinez et al., 2015, 2017; Parker et al., 2021). The ecological conditions, such as the host’s nutritional requirements (Holland et al., 2004; Rio et al., 2006; Simonet et al., 2016; Vigneron et al., 2014), diet (Snyder et al., 2012; Wilkinson et al., 2007; Zhang et al., 2016), infection status (Scarborough et al., 2005) and temperature (Russell & Moran, 2006), also influence the net benefit of the relationship for the host.

A general theoretical model of mutualisms predicts that the ability to control the population size of a symbiotic partner in response to the ecological conditions could provide a fitness advantage to the host, as the symbiont density could be continually adjusted to that which maximizes the net benefit for the host (Holland et al., 2004). Indeed, there is extensive empirical evidence to suggest that animals, such as insects and corals, actively regulate (i.e. control and adjust) the density of their symbionts according to their requirements (Cunning et al., 2015; Feldhaar et al., 2007; Hamidou Soumana et al., 2013; Kodama & Fujishima, 2012; Lowe et al., 2016; Rio et al., 2006; Simonet et al., 2016; Snyder et al., 2012; Vigneron et al., 2014; Whittle et al., 2021; Wilkinson et al., 2007; Wolschin et al., 2004; Zhang et al., 2016). Two features of host-symbiont associations likely facilitate such active host regulation of symbiont density. First, compartmentalization of symbionts within specialized host tissues is a widespread characteristic of obligate associations between hosts and their vertically transmitted symbionts, and can allow hosts to control the resources made available to the symbionts (Chomicki et al., 2020). Second, symbionts involved in highly intimate and ancient mutualisms demonstrate significant genome decay (Akman et al., 2002; Gil et al., 2003). Such reduced genomes in symbionts suggest loss of autonomy and specifically a reduced ability of these symbionts to regulate their own proliferation.

Recent discussion has highlighted that forming obligate associations with hosts is not necessarily beneficial for symbionts, for example, due to increased risk of extinction and the fixation of deleterious mutations (Garcia & Gerardo, 2014; Keeling & McCutcheon, 2017). The effect of host association on symbiont fitness has been studied elsewhere (e.g. (Lowe et al., 2016)), however, the effect of host-controlled regulation of symbiont density (in comparison to unregulated symbiont growth) has, to our knowledge, not been explored. Investigation of symbiont regulation can be difficult to approach empirically, due to the inability to measure simultaneously within-host symbiont density and the direct effect this has on an individual host. Additionally, current methods prevent us from culturing many intracellular symbionts outside of their hosts, rendering these very intimate symbioses challenging to manipulate experimentally. Studies comparing the consequences of symbiont density for hosts and symbionts require comparing symbionts of different strains which are naturally present in their hosts at differing density (Chong & Moran, 2016; Martinez et al., 2015). However, differences in symbiont and host genotype may have unknown effects. In lieu of feasible experimental procedures, theoretical models can be developed to investigate the underlying ecological drivers and fitness outcomes of symbiont regulation. The advantage of this approach is that we can predict the general evolutionary interactions for a range of symbiotic partners. Moreover, by tailoring the details of the interactions to specific symbioses, we can generate testable predictions for experimentation.

Here, we construct a general framework for modelling symbiont density regulation in a mutualism between an iteroparous host (i.e., one which reproduces on a regular basis from the start of adulthood) and an obligate symbiont which is compartmentalized within the host and transmitted vertically to the host’s off-spring (Baumann et al., 2013; Buchner, 1965; Dubilier et al., 2008; Nowack & Melkonian, 2010). Several mechanisms are used by hosts to control their symbiont populations, for example, immune factors (Anselme et al., 2008; Ratzka et al., 2011), digestion (Nishikori et al., 2009; Vigneron et al., 2014) and expulsion (Baghdasarian & Muscatine, 2000; Fishman et al., 2008) to reduce the number of symbiont cells. For simplicity, in our framework, hosts control the proliferation of their symbiont via the allocation of dietary energy (Bever et al., 2009; Dean et al., 2016; Graf & Ruby, 1998; Lowe et al., 2016). The framework assumes that greater symbiont densities are more energetically costly to maintain, but also provide greater benefits to the host (Chong & Moran, 2016; Chrostek et al., 2013; Cunning & Baker, 2014; Drew & King, 2022; Martinez et al., 2015, 2017; Parker et al., 2021). Hosts therefore experience a trade-off between the investment made in supporting a symbiont population, and the energetic resources kept in reserve for maintaining other aspects of host biology, such as somatic repair and reproduction.

First, we use dynamic optimization to predict the optimal strategy of energy allocation to a symbiont population, i.e., that which maximizes host fitness, building on the assumption that symbiont regulation is an active and adaptive host behaviour. We then simulate symbiont density throughout host development for hosts employing the optimal strategy. Using a general framework which is not parameterized to a particular host-symbiont system, we use a sensitivity analysis to assess the effect of individual parameters on the emergent dynamics of symbiont density. Furthermore, we explore host-controlled symbiont regulation by addressing the following questions:

- How are the dynamics of symbiont density over the course of an individual host’s development affected by perturbation in both symbiont density and host energy reserves from their stable values back to starting levels at set points in development?
- How do the fitness consequences (to hosts and symbionts) of the optimal energy allocation strategy compare to those of the following four alternative regimes of energy allocation: imperfect implementation of the optimal strategy; fixed allocation; allocation proportional to symbiont density; and the strategy which maximizes symbiont fitness?

Finally, we demonstrate the versatility of such a modelling approach by adapting the base-case model to predict trends in symbiont regulation for symbioses with different ecologies:

- non-essential symbionts,
- symbionts which are not required for survival after host maturation, and
- hosts which experience extended post-reproductive life.

We discuss the results in light of recent developments in the evolutionary ecology of host-symbiont interactions.

## 2 Methods

We used dynamic programming in a state-dependent model to determine the optimal host strategy of energy allocation to an obligate, vertically transmitted symbiont, throughout host development (Houston et al., 1988; Houston & McNamara, 1999).

The state of the host is defined by two state variables: the host’s energy reserves (*E*) and the within-host symbiont density (*W*). The model contains discrete time steps (*t*), each corresponding to the host taking a meal. At each time step, the host makes one decision: how much energy to invest into the symbiont population, which changes in density according to this decision. The optimal decision made by the host is based on its current state, being that which maximizes the lifetime fitness of the host (i.e. the total reproductive success of distant descendants).

We built the base-case model for a female host reproducing every four time steps, with the first reproductive event occurring at *t* = 8. The choice for the reproductive schedule was arbitrary and alternative schedules were considered in the Supporting Information (Table S1, Figures S1, S2 and S3). Table 1 shows the model parameters and default values used in the base-case model.

**Figure 1:**
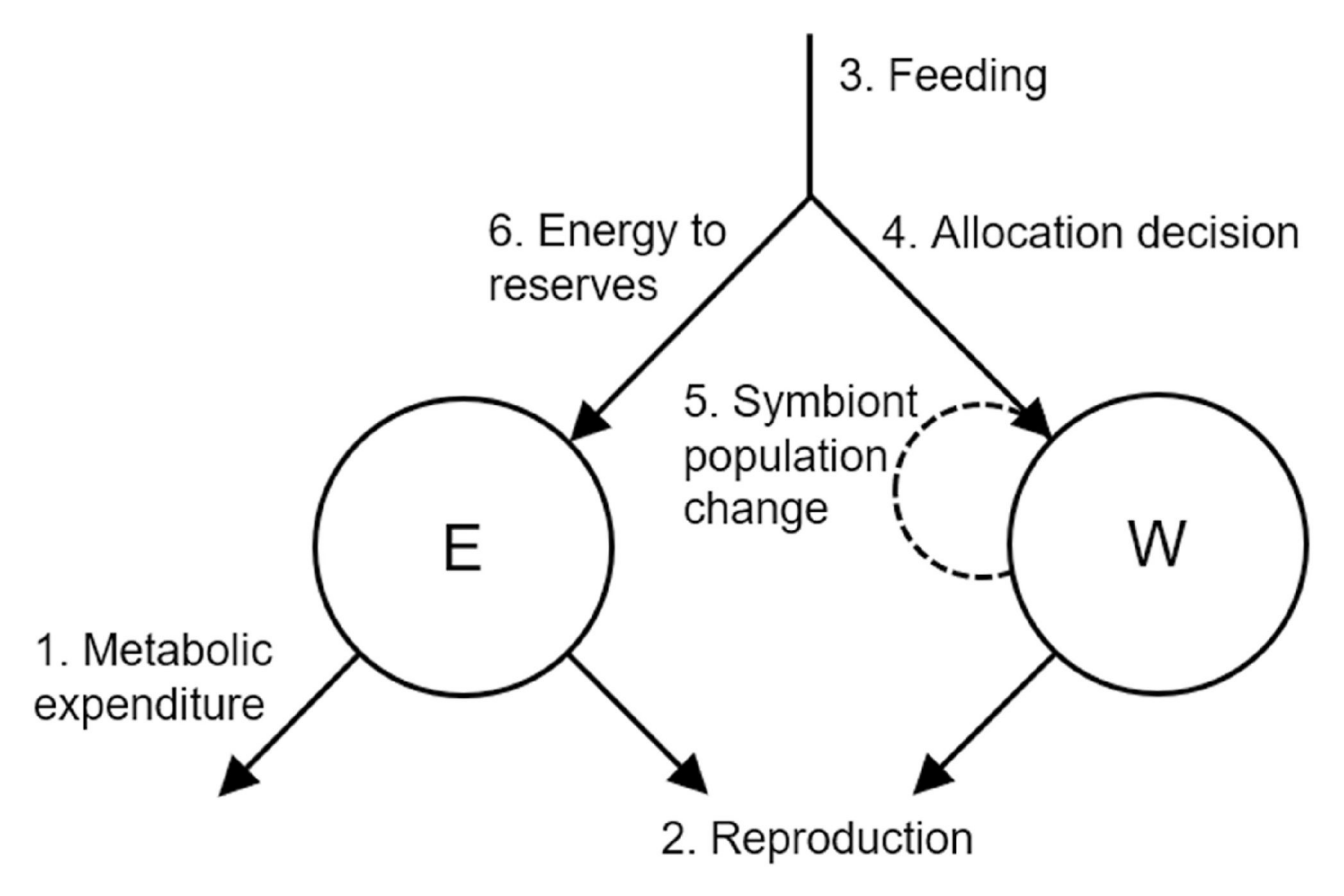
The order of events which occur at each time step. At the start of time step *t*, the state of the host is determined by the host’s energy reserves, *E*(*t*), and symbiont density, *W*(*t*).

At the start of time step *t*, the energy reserves are equal to *E*(*t*) and the density of symbiont population is equal to *W*(*t*). During each time step, the order of events are as follows (see Figure 1 for a schematic):

(1) Energy is lost from the host’s energy reserves through background metabolic expenditure (*E*_exp_), which scales linearly with the host’s reserves:
  $$\begin{array}{l} E^{\prime }(t)=E(t)-E_{\text{exp}}\\ E_{\text{exp}}=\mu E(t)+\omega \end{array}$$
  where *µ* is the proportional increase in metabolic expenditure with energy reserves and *ω* the minimum expenditure (Table 1).
(2) The host reproduces, if it is a reproductive time step and the host has sufficient energy reserves and symbiont density, dependent on threshold levels *E*_crit_ and *W*_crit_ respectively, i.e.:
  $$\begin{array}{c} E^{\prime (t)}\geq E_{\text{crit}\;}\\ W(t)\geq W_{\text{crit}.\;} \end{array}$$
  During reproduction, a proportion (*λ*) of the host’s current energy reserves are invested into reproduction (*E*_rep_):
  $$\begin{array}{c} E_{\text{rep}\;}=\lambda E^{\prime }(t)\\ E^{\prime \prime }(t)=E^{\prime }(t)-E_{\text{rep}\;} \end{array}$$
  A proportion (*γ*) of the symbiont population within the host is also transferred vertically to the offspring (*W*_rep_):
  $$\begin{array}{c} W_{\text{rep}\;}=\gamma W(t)\\ W^{\prime }(t)=W(t)-W_{rep\;} \end{array}$$
(3) The host gains energy through feeding, equal to *N*. The value for *N* is fixed at every time point, as stochasticity in feeding does not significantly affect the results (Figures S4, S5).
(4) The host makes the decision *u*: how much dietary energy should be allocated to the symbiont population? The available actions are all values:
  0≤u≤N
(5) The symbiont population adjusts according to the allocation decision *u*. At the start of the next time step, the density of symbiont is given by:
  $$W(t+1)=W^{\prime \prime }(t)=\{\begin{array}{c} W^{\prime }(t)+\left(u-\alpha W^{\prime }(t)\right)\beta ,\;W^{\prime }(t)>0\\ 0,W^{\prime }(t)=0 \end{array}$$
  where *α* is the cost of maintaining the symbiont and *β* is the amount of symbiont growth per unit of energy (Table 1).
(6) The remaining dietary energy is added to the host’s reserves. At the start of the next time step, the level of energy reserves is therefore:
  $$E(t+1)=E^{\prime \prime \prime }(t)=E^{\prime \prime }(t)+N-u$$

### 2.1 Dynamic optimization

The dynamic programming equation describes the reproductive value of hosts as determined by their current state and allocation decision:

H(E(t),W(t);u)=B(E(t),W(t);u)+S(E(t),W(t);u)V(E(t+1),W(t+1))

where

*H*(*E*(*t*), *W*(*t*); *u*) is the reproductive value of the host in state *E*(*t*) and *W*(*t*), given the decision *u* and

*B*(*E*(*t*), *W*(*t*); *u*) is the immediate contribution to fitness of a host in state *E*(*t*) and *W*(*t*), given decision *u*:

$$B(E(t),W(t);u)=E_{\text{rep}\;}W_{\text{rep}\;}$$

We assume linear dependence of *B* on *E* and *W*. Other functional forms were considered in the supplementary material (Table S2, Figure S6). The immediate contribution to fitness is calculated at the end of the time step.

*S*(*E*(*t*), *W*(*t*); *u*) is the probability of survival from *t* to *t* + 1 of a host in state *E*(*t*) and *W*(*t*), given decision *u*:

$$S(E(t),W(t);u)=\left(1-e^{-0.3E^{\prime \prime \prime }(t)}\right)\left(1-e^{-0.3W\prime \prime (t)}\right)$$

Survival is assumed to increase asymptotically with both *E*(*t*) and *W*(*t*). We selected the exact form for the dependence of survival on energy reserves and symbiont density as that which maximized host longevity without reaching the time horizon (*T*).

*V*(*E*(*t* + 1), *W*(*t* + 1)) is the reproductive value of the host at time *t* + 1, given that the host behaves optimally from *t* + 1 to *T*. The state given by *E*(*t* + 1) and *W*(*t* + 1) is the new state of the host at *t* + 1. At the time horizon, the terminal reproductive value is given by the immediate contribution to fitness:

V(E(T),W(T))=B(E(T),W(T);u)

For a host in state *E*(*t*) and *W*(*t*) at time *t*, the optimal decision (u*) is that which maximizes the reproductive value:

$$H\left(E(t),W(t);u^{*}\right)={\text{max}}_{u}[H(E(t),W(t);u)]$$

The optimal decision for each value of *E, W* and *t* was obtained by iterating backwards from *t* = *T* to *t* = 1, where

$$V(E(t+1),W(t+1))=H\left(E(t),W(t);u^{*}\right)$$

### 2.2 Forward simulations

We used forward simulations to ascertain the effect of the optimal host regulation strategy on measurable symbiont density. To gain a representation of the symbiont dynamics from a host population containing all possible phenotypes, we simulated ten hosts for each possible state at *t* = 1 (4000 total). The lifetime fitness for each host simulation (*V_H_*) was calculated by the sum of all immediate contributions to host fitness:

$$V_{H}=\underset{t}{\sum \;}B(t)$$

The total fitness of the symbiont (*V_S_*) was calculated by the sum of all symbiont density transferred to offspring over the course of host lifetime:

$$V_{S}=\underset{t}{\sum \;}W_{\text{rep}\;}(t)$$

### 2.3 Sensitivity analysis

We analysed the sensitivity of the dynamics of symbiont density throughout host development to specific parameters (Table 1) using Latin hypercube sampling of the parameter space (Saltelli et al., 2008). The range for each selected parameter was determined by the limits which produced models where host fitness was non-zero and states were not maximized, while all other parameters were at their default value. For use in correlation analysis, we approximated the level at which the symbiont density plateaued by averaging the symbiont density over all reproductive time steps. We then found the linear correlation between each parameter and the average symbiont density using Pearson’s correlation (Table 2).

### 2.4 Exploration of the optimal strategy

First, we investigated the effect of perturbations to the host’s state during host development on symbiont density. Specifically, we simulated the subsequent dynamics of symbiont density for hosts of all states when levels of both symbiont density and energy reserves were shifted from their stable states to the starting values at random time steps: *t* = 7, *t* = 24 and *t* = 39.

Second, we compared the optimal (base-case) strategy to alternative patterns of energy allocation, by generating simulations for:

(i) Hosts which imperfectly implemented the optimal strategy of energy allocation, i.e., at each time step the allocation (*u*) was sampled from a continuous uniform distribution:
  $$u∼U\left(u^{*}-1,u^{*}+1\right)\;\text{constrained}\;\text{to}\;0\leq u\leq N$$
(ii) Hosts which allocate a fixed amount of energy (*u* = 3), regardless of their state and time step. *u* = 3 was selected from the range 1 – *N* as this value resulted in the greatest average fitness for both hosts and symbiont.
(iii) Hosts which used the strategy which maximizes symbiont fitness, given by the amount of symbiont transferred to offspring only (Table 3):
  $$B(E(t),W(t);u)=W_{\text{rep}\;}$$
  This strategy was found by backwards iteration of the dynamic programming equation.
(iv) Hosts which transferred an amount of energy proportional to the symbiont population:
  u=0.3Wwith0≤u≤N
  0.3 was selected from the range 0.1–1 as this proportion resulted in the greatest average fitness for both symbiont and hosts.

We conducted Kruskal-Wallis tests to examine differences in fitness (for hosts and symbionts) from simulations using the different strategies of regulation. Significant differences in pairwise interactions were then determined using Wilcoxon Rank Sum tests. We used a significance criterion of 5% for all statistical tests.

### 2.5 Expansions of the base case model to alternative host-symbiont ecologies

Finally, we generated the optimal strategy for energy allocation and resulting simulations for three additional host-symbiont ecologies beyond that considered in the base-case model:

- For a non-essential symbiont, characterized by reduced influence on survival and fitness, and no minimum symbiont density required for reproduction (Table 3).
- For a symbiont where, after reaching maturation, the probability of survival was independent of the symbiont density (Table 3).
- For a host with a post-reproductive lifespan, i.e., where the final reproductive cycle takes place before the time horizon (Table 3).

## 3 Results

### 3.1 Base-case model

**Figure 2:**
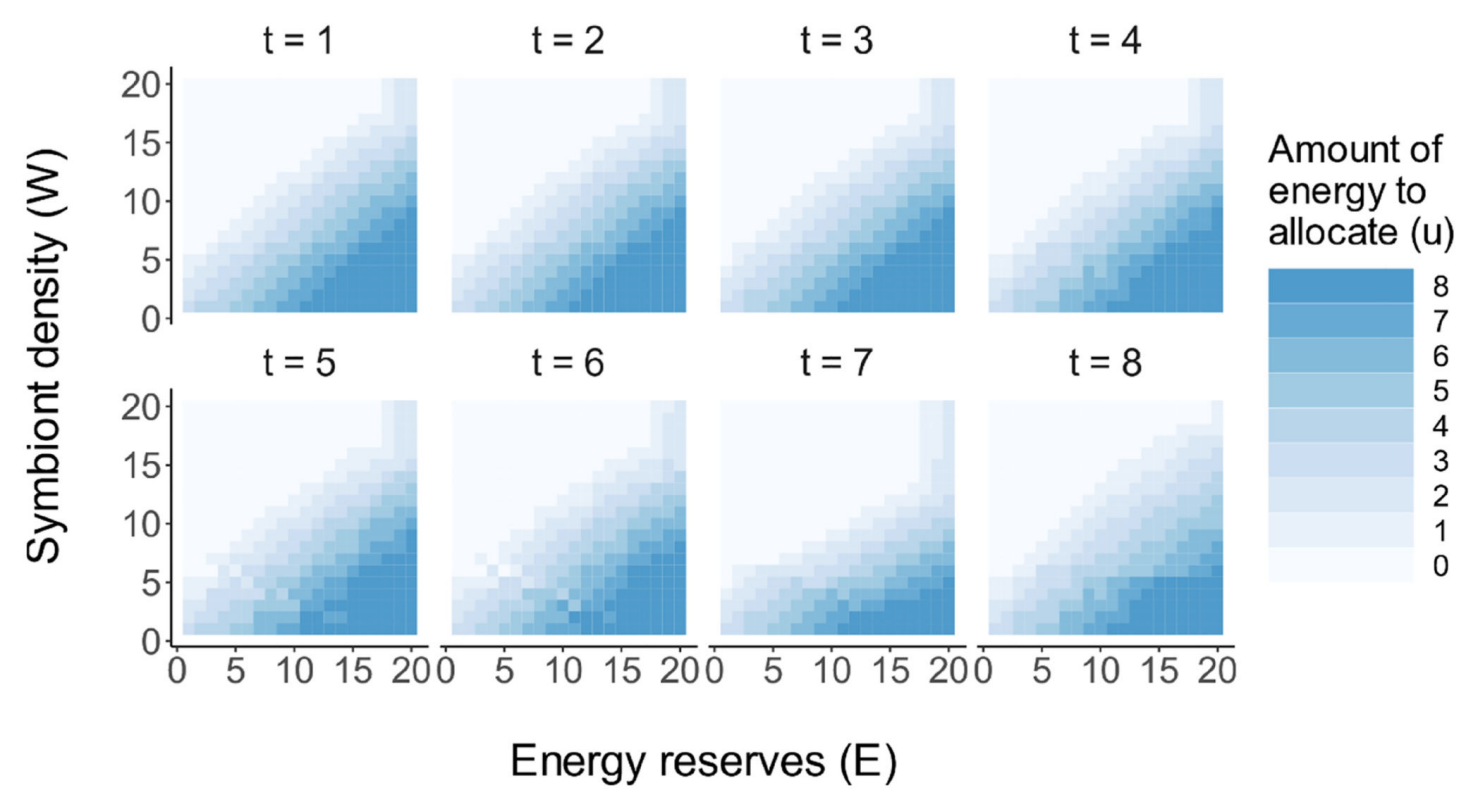
The optimal amount of energy for a host to allocate to the symbiont population dependent on its energy reserves and symbiont density, for selected time steps. The strategy for *t* = 5 to *t* = 8 repeats throughout host development.

The optimal allocation of energy to the symbiont population depends both on host energy reserves *E* and symbiont density *W* (Figure 2). Below, we present model output from the first eight time steps as the strategy repeats every four time steps (concurrent with the reproductive cycle) after *t* = 8. In general, the optimal strategy from the base-case model dictates that all of the dietary energy obtained in one meal (*N* = 8) should be allocated to the symbiont population when energy reserves are very high and symbiont density is very low, and this is consistent throughout host development. The maximum possible growth of the symbiont is a 425% increase in symbiont density following a non-reproductive time step. When the symbiont density is high and energy reserves are not sufficient to support the population, energy should be withheld completely, equating to a 25% reduction in symbiont density (following non-reproductive time steps).

**Figure 3:**
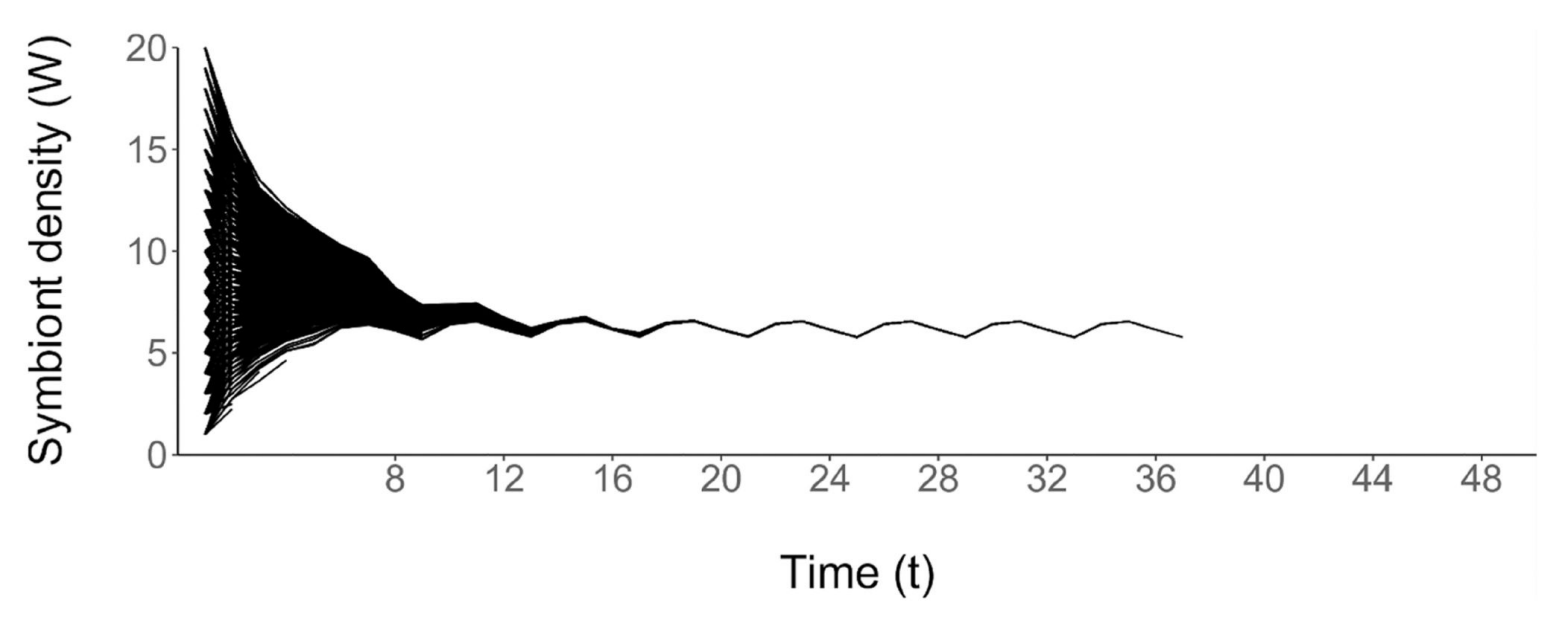
Symbiont density throughout host development, subjected to the optimal strategy of host regulation (ten hosts per state at *t* = 1, 4000 total). Each line indicates the symbiont density of one simulated host. The time steps of reproductive events are indicated on the x-axis.

Between *t* = 0 and the onset of reproduction at *t* = 8, the range in symbiont density of the simulated hosts is reduced from 19 (i.e., 1–20) to 2.1 (6.1–8.2), indicating that symbiont density is rapidly regulated to an optimal level (Figure 3). For hosts which begin life with low levels of symbiont density, the optimal strategy of energy allocation results in symbiont population growth prior to the first reproductive event. In contrast, for hosts which begin life with high levels of symbiont density, energy is withheld completely from the symbiont population to allow it to reduce in size. From the time of the first reproduction (at *t* = 8), the symbiont density is maintained around an average level of *W* ≈ 6.3 (Figure 3). The allocation of energy cycles between *u* ≈ 2.4 and *u* ≈ 4.9 units of energy, concurrent with the reproductive cycle. Symbiont density reaches a local maximum shortly before reproduction, and reaches a local minimum post-reproduction. Once the persisting dynamic is established, the energy allocated at each time step allows the symbiont to increase in density, with vertical transmission to the host offspring preventing continual growth of the symbiont population. As such, the symbiont density of all simulated hosts converges on a stable oscillation between *W* ≈ 5.7 and *W* ≈ 6.6. Alternative reproductive schedules did not produce qualitatively different results (Figures S1, S2, S3).

### 3.2 Sensitivity analysis

Ten model parameters are correlated with the level around which the symbiont density plateaued (Table 2), five of which had significant effects. Specifically, the level at which *W* plateaued is moderately and positively correlated with the intake of dietary energy (*N*) and the amount of symbiont density created per unit of energy (*β*), and negatively correlated with the background metabolic expenditure of hosts (*µ* and *ω*) and the metabolic cost of maintaining symbionts (*α*). Five other parameters were correlated but appeared to have no significant effect: the time horizon (*T*), the minimum energy reserves required to reproduce (*E*_crit_), the minimum symbiont density required to reproduce (*W*_crit_), the proportion of energy reserves invested into reproduction (*λ*) and the proportion of symbiont density transmitted to offspring (*γ*).

### 3.3 Exploration of the optimal strategy

Changes in host state which occur during host development result in the symbiont density rapidly returning to the level prior to perturbation (Figure 4), indicating that this dynamic is stable to perturbations in the energy reserves or symbiont density of the host. This convergence occurs for all magnitudes of perturbation in both energy reserves (*E*) and symbiont density (*W*), and at each of the random time points *t* = 7, 24 and 39 when the perturbation occurs.

**Figure 5:**
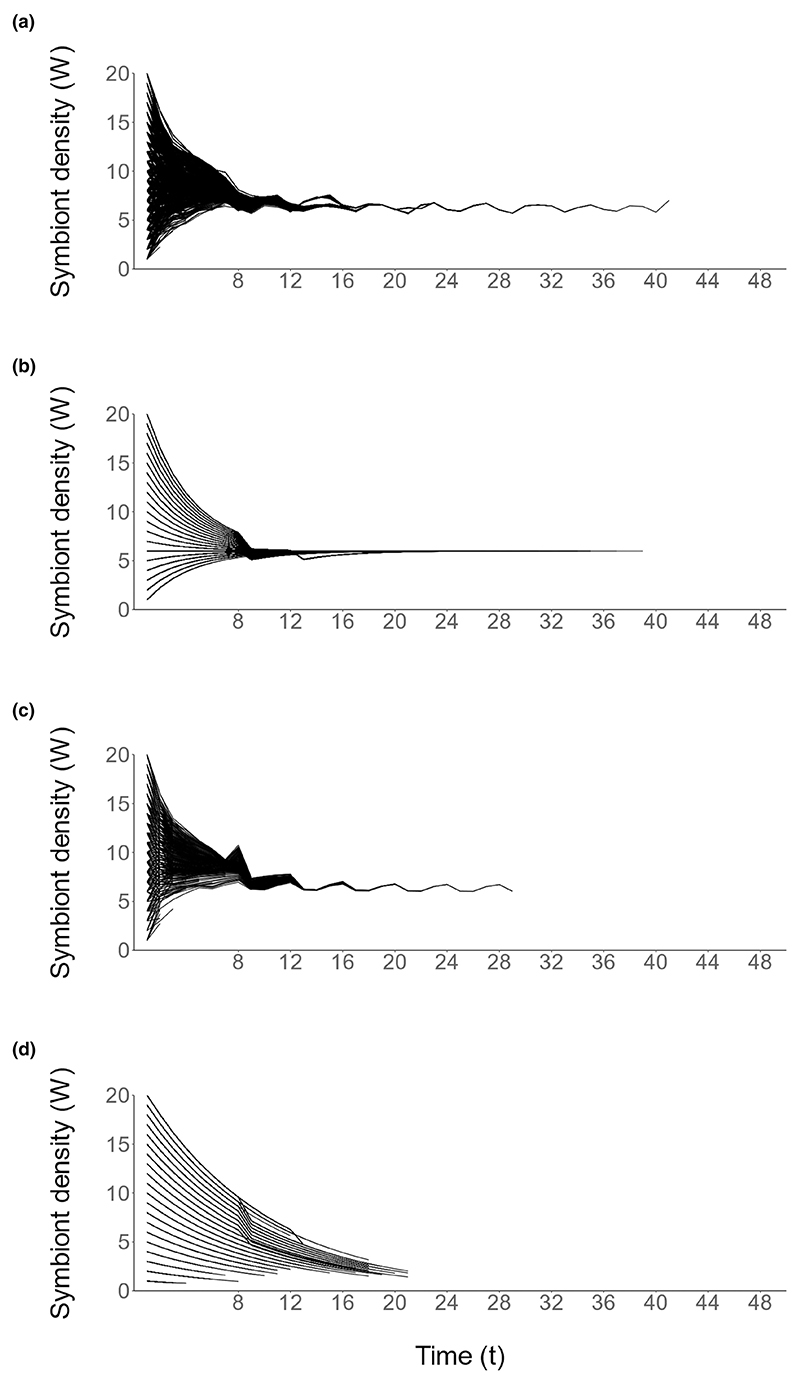
Symbiont density of hosts employing alternative energy allocation strategies (ten hosts per state at *t* = 1, 4000 total) (a) Hosts imperfectly executing the optimal strategy, i.e. allocating an amount of energy sampled from a uniform distribution around the optimal allocation decision (*u* ∼ *U*(u* − 1, u* + 1)) (b) Hosts allocating a fixed amount of energy (*u* = 3) at each time step, regardless of host state (c) Hosts employing the strategy which maximizes symbiont fitness (d) Hosts allocating an amount of energy proportional to the level of the symbiont density at every time step, i.e., *u* = 0.3 *W*, regardless of host energy reserves.

Three of the alternative strategies of energy allocation produce symbiont density dynamics similar to the optimal strategy, whereby the density converges on a plateau around *W* ≈ 6 (Figure 5). The energy allocation regime which did not produce this trend was the allocation of an amount of energy proportional to the symbiont density, whereby symbiont density was depleted in all hosts and survival reduced (Figure 5d).

**Figure 6:**
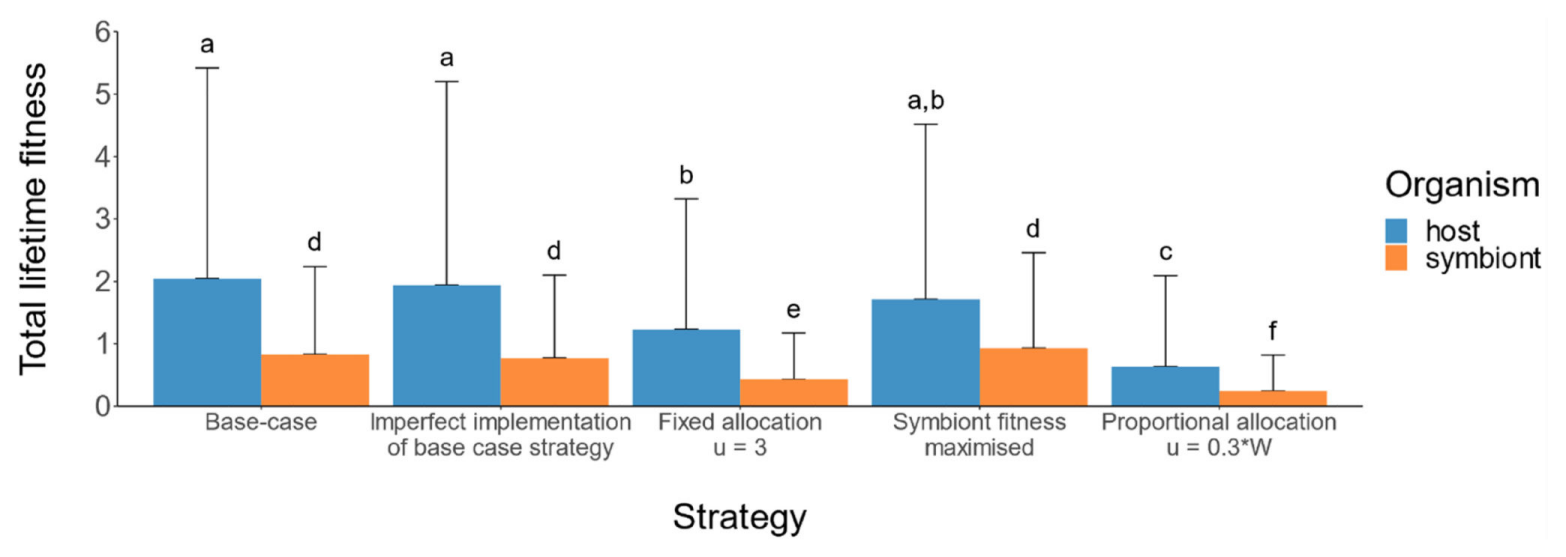
Total lifetime fitness for hosts (*V_H_*), blue, and symbionts (*V_S_*), orange, from simulations, under the base-case and four alternative energy allocation strategies (*n* = 4000 simulations per strategy). Means are represented and error bars signify 1 SD. Letters indicate significant differences between strategies (*p* < 0.05).

There were significant differences in host fitness (*V_H_*, H(4) = 500.96, *p* < 0.05) and symbiont fitness (*V_S_*, H(4) = 528.44, *p* < 0.05) between the strategies (Figure 6). There were also significant pairwise interactions between strategies, the results of which are shown in Table 4.

### 3.4 Expansions of the base-case model to alternative host-symbiont ecologies

**Figure 7:**
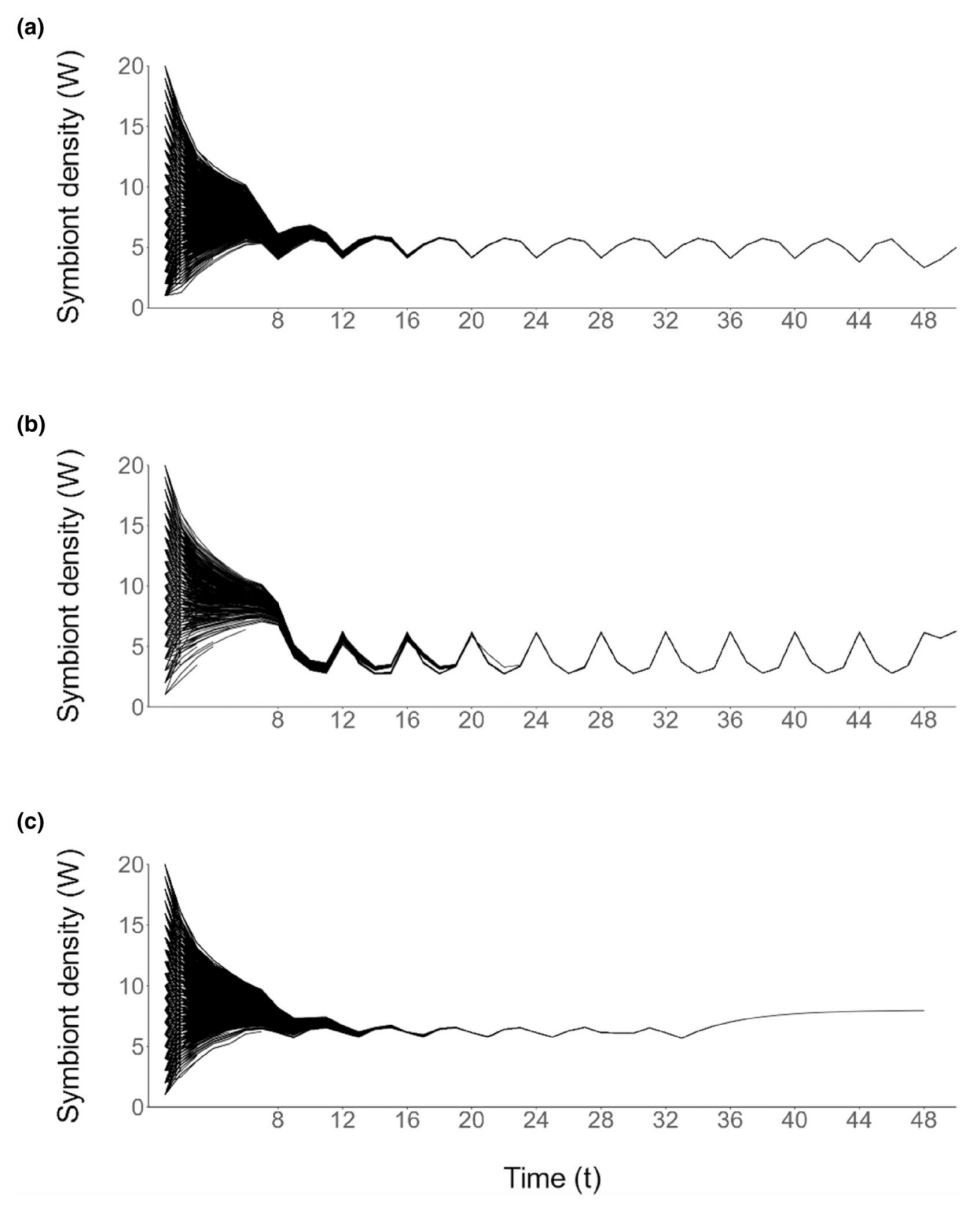
Symbiont density for expansions of the base-case model to different host-symbiont ecologies (Table 3, ten hosts per state at *t* = 1, 4000 total). (a) For a non-essential symbiont (b) For a symbiont which is no longer required for survival after host maturation. (c) For hosts which demonstrate post-reproductive life.

The dynamics of symbiont density for the three other host-symbiont ecologies – i.e., non-essential symbionts, symbionts only required during host maturation, and hosts with extended post-reproductive lifespan – demonstrate some similar trends to the base case, whereby the symbiont oscillates around a particular density during the reproductive period of their hosts’ life and the symbiont density converges onto the same dynamic for all host states (Figure 7).

Non-essential symbionts are maintained at a lower density (≈ 3.8) than that of the base-case (Figure 3), and the oscillations in symbiont density during the reproductive cycle are greater, whereby symbiont density fluctuates between *W* ≈ 2.9 just after reproduction and *W* ≈ 5 just prior to reproduction (Figure 7a).

For a symbiont with temporary benefits, the distribution of symbiont density is similar to the base-case during maturation (*t* > 8), and depleted at the onset of reproduction (Figure 7b). While the symbiont is no longer needed for survival, it is maintained for transmission to host offspring, increasing in density up to *W* ≈ 6.3 prior to reproduction and then returning to the base level of *W* ≈ 3.9.

For hosts with post-reproductive lifespans, the allocation of energy is such that the symbiont proliferates and converges on a new level of symbiont density after the final reproductive event, which occurs at *t* = 32 (Figure 7c).

## 4 Discussion

Here, we have investigated host-controlled regulation of the density of an obligate, vertically transmitted symbiont using a mathematical modelling approach. Specifically, we used dynamic programming to explore how resource allocation decisions and different ecological scenarios affect symbiont density regulation. Our model produces general results which could be used to set some empirical studies in context (given the challenges in explicitly measuring or manipulating symbiont density and host ecology), as well as providing a basis to be adapted to more tailored models applied to specific systems.

We find that the density of symbiont subjected to state-dependent regulation by its host converges on the same dynamic for all hosts (Figure 3), indicating that maintaining a moderate symbiont density throughout development is optimal for a host to maximize its fitness, regardless of the energy level and symbiont density at the onset of development. The simulations suggest that, first, symbiont density rapidly converges for hosts of different starting states, and, second, symbiont density is stable to changes in host state. Similar trends have been reported in studies on tsetse (*Glossina morsitans morsitans*), where little variability was reported in the density of the symbiont, *Wigglesworthia*, between hosts of the same age and sex, and elevated levels of symbiont density are found in the offspring of stressed females at birth, before returning to levels similar to controls (Rio et al., 2006). Similarly, the density of dinoflagellate symbiont in *Pocillopora damicornis* coral has been shown to converge when transferred to constant environmental conditions (Cunning et al., 2015).

The results of the sensitivity analysis indicate that the approximate level around which the symbiont density converges is significantly and negatively correlated with the parameters determining the background metabolic expenditure of the host (*µ* and *ω*) and positively correlated with the dietary intake of energy (*N*). As the metabolic expenditure of the host increases, there is less energy available for allocation to the symbiont, therefore the symbiont is maintained at a lower level. Likewise, low symbiont densities are maintained when there is less energy available in the diet of the host. The model assumes a simple mechanism of symbiont regulation, whereby the energy allocated to the symbiont population is fully controlled by the host. Other regulatory mechanisms, such as immune factors (Anselme et al., 2008; Ratzka et al., 2011), require an energy investment which may result in a different relationship between energy availability and the optimal density of symbiont. The influence of host diet on symbiont density is well reported (e.g. (Snyder et al., 2012; Zhang et al., 2016)), and experiments whereby aphids (*Acyrthosiphon pisum*) are reared with varying availability of dietary nitrogen have demonstrated that hosts with nitrogen-rich diets harbour larger populations of their symbiont, *Buchnera* (Wilkinson et al., 2007). We predict that rearing hosts with a reduced energy content will also result in lower symbiont densities in comparison to hosts receiving a full diet. The parameters which determine how the symbiont uses the allocated energy are also significantly correlated with the level at which symbiont density plateaus: this correlation is negative for the maintenance cost of the symbiont (*α*), and positive for the increase in symbiont density per unit of energy (*β*).

The parameters which do not relate to energy availability or use are not significantly correlated with the level at which symbiont density plateaus. Of particular interest, there was no significant correlation with the proportion of symbiont transmitted to offspring (*γ*). It may be that it is not sufficiently costly to increase the symbiont density, hence whether a small or large amount of symbiont is lost during transmission has little effect on the optimal symbiont density as it can be easily replenished before the next reproductive event. Rapid proliferation of symbionts following initial colonization has been reported in immature hosts (Rio et al., 2006; Ruby & Asato, 1993; Simonet et al., 2016; Wolschin et al., 2004), however it is unknown what cost this imposes on the host and if this rapid growth would occur in mature hosts. The consequences of symbiont proliferation could be determined by experimentally reducing symbiont density in mature hosts, then measuring the rate at which the density returns to previous levels and the impact this has on survival and reproduction. We anticipated that the minimum symbiont density required to reproduce (*W*_crit_) would correlate with the level at which symbiont density plateaus; however, the Latin hypercube sampling method used in the sensitivity analysis means that the other parameters are not fixed at their default values for each model, so only the parameters with the strongest influence (i.e. independent of other parameter values) produce significant correlation with the level at which the symbiont density plateaus.

The dynamics of symbiont density which emerge from regulation according to some alternative energy allocation strategies (Figure 5), for example, allocating a fixed amount of reserves to symbionts, also plateau around the onset of reproduction. Such a trend observed empirically would therefore not be conclusive evidence of state-dependent regulation by the host. However, the fitness of both hosts and symbiont is significantly reduced when a fixed amount of energy is allocated to the symbiont or when energy is allocated proportional to symbiont density (Figure 6), indicating that a state-dependent allocation strategy is adaptive for both parties. Both host and symbiont perform well regardless of whether the strategy of regulation is optimized to maximize host or symbiont fitness (Figure 6), indicating that their fitness interests are aligned. Symbiont genome decay is a characteristic of ancient associations between obligate symbionts and their hosts (McCutcheon & Moran, 2011), one effect being that several well-characterized symbionts have been reported to be missing the mechanisms necessary for autonomous self-replication (Akman et al., 2002; Gil et al., 2003; Kogoma, 1997). Our results suggest that while subject to host-controlled regulation, the inability for symbionts to regulate their own density is not necessarily deleterious for the symbiont, and selection on symbionts to maintain their regulatory systems for self-replication could therefore become relaxed. There is no statistically significant difference in the fitness of either hosts or symbiont when the base-case strategy is imperfectly implemented (Table 4), indicating also that there could be relaxed selection pressure on hosts to achieve optimality.

The expansions of the model beyond the base case highlights the tractability of this modelling framework for predicting the symbiont density dynamics for various ecologies of compartmentalized symbionts. The general trend of periodic allocation of energy concurrent with the reproductive cycle is demonstrated for all scenarios (Figure 7). However, the oscillation in symbiont density is variable. When the symbiont is no longer required for survival, it is maintained at a low level and greatly increased just prior to reproduction (Figure 7b). The density of symbiont *Blochmannia* has been shown to decrease in non-reproductive female carpenter ants (*Camponotus floridanus*) after reaching maturation (Wolschin et al., 2004). However, after isolation from the rest of the colony, previously non-reproductive females begin producing offspring, and such females harbour greatly increased symbiont densities (Wolschin et al., 2004). This supports our prediction that hosts may reduce the density of symbionts when they are no longer required for survival, but will increase the density during reproduction to maximize the benefit of the symbiosis for their offspring.

Our simulations predict that the symbiont density will increase if host reproduction ceases during adulthood (Figure 7c). This trend is observed in unmated female tsetse, which demonstrate sustained symbiont growth during adulthood (Rio et al., 2006), and could be the result of more energy being available to the symbiont population as it is not required by the host for reproduction. This contrasts with data collected from parthenogenic aphids (*A. pisum*), which demonstrate a depletion in *Buchnera* density after the laying period (Simonet et al., 2016), suggesting that aphids reduce the symbiont population as they no longer require symbionts at a high density. One limitation of our model is that energy intake is constant throughout host development. However, due to hosts no longer investing large amounts of energy on reproduction, energy intake may reduce in post-reproductive hosts. The amount of energy available for allocation to the symbiont population may therefore be less than predicted by our results. This warrants further investigation.

The generality of our modelling framework limits the precision with which it captures any specific host-symbiont biology. First, autophagy and apoptosis of symbiont housing cells have been shown to actively reduce symbiont populations in weevils and aphids (Nishikori et al., 2009; Simonet et al., 2018; Vigneron et al., 2014). Such immune functions would require an energetic investment from the host, and digestion of symbiont cells could return energy to the host (Vigneron et al., 2014). While our modelling framework does not account for direct removal of symbiont cells, modelling symbiont density regulation via energy allocation is an appropriate simplification for compartmentalized symbionts as it broadly captures the energetic trade-off experienced by hosts. While bacterial growth under a constant supply of energy has been modelled extensively (e.g. (Contois, 1959)), given the apparent lack of autonomous self-replication characteristic of symbionts from ancient symbioses (McCutcheon & Moran, 2011), our simple model of symbiont density adjustment due to energy allocation from the host is justified. Furthermore, our framework assumes linear dependence of host fitness on symbiont density, but this is a simplification as it is not likely that the costs and benefits will continue to increase at very large symbiont densities (Drew & King, 2022; Parkinson et al., 2017). However, when alternative functions forms for *B*(*E*(*t*), *W*(*t*); *u*) were tested, qualitatively similar results were produced (Figures S7–S14). Obligate association with a host renders symbiont fitness challenging in terms of theory and empirical testing. Here, we calculate symbiont fitness (*V_S_*) by the total amount of symbiont density which is inherited by the host’s offspring, however, other factors beyond the scope of this study, such as transmission fidelity and genetic bottlenecks, will impact symbiont fitness on an evolutionary timescale (Bennett & Moran, 2015).

Here, we consider one symbiont population, housed within specialized organs or cells which allow the host to control the availability of energy to the symbiont. Some hosts have been shown to harbour a secondary symbiont population for transmission to offspring. For example, female tsetse harbour the main population of their symbiont (*Wigglesworthia*) in a specialized bacteriome just off the gut, yet transmit *Wigglesworthia* to their developing larvae in a milk-like substance, from a secondary population contained within the reproductive tract (Balmand et al., 2013). The strategy and mechanism by which hosts should regulate this secondary population remains unexplored. Additionally, examples are known of horizontally transmitted symbionts which are subject to a high degree of control by their host (Kim et al., 2016), and adaptation of the modelling framework could expand our understanding of symbiont regulation in these alternative systems. Furthermore, hosts appear to transmit varying amount of symbiont to their offspring depending on their physiological conditions, e.g. stress levels (Rio et al., 2006) and age (Hosokawa et al., 2007). Whether and how the amount of symbiont being transmitted to offspring is actively regulated by the parent is unknown and warrants further investigation. A similar framework to that developed herein could be used for such investigation.

## 5 Conclusions

We use dynamic programming to predict the optimal strategy for hosts when regulating the density of an obligate, vertically transmitted symbiont via the allocation of energy. Simulations of hosts using this optimal strategy of regulation throughout their development suggest that hosts should regulate their symbiont density around a constant level, and this dynamic is robust to perturbation in host state. Ecological factors which affect the intake or energy usage influence the optimal level around which the symbiont density should be maintained. The optimal energy allocation strategy promotes symbiont fitness in comparison to alternative strategies, which suggests that active host-controlled regulation of symbiont density could be adaptive for the symbiont as well as the host. We demonstrate the versatility of our theoretical approach for investigation into the evolutionary ecology of host-endosymbiont systems by adapting the framework for predicting symbiont density dynamics for various host-symbiont ecologies, such as non-essential symbionts and hosts which experience a post-reproductive lifespan. This study demonstrates a novel approach to investigating the governing forces underlying highly intimate symbiotic interactions between hosts and their mutualistic microbes, by applying dynamic optimization modelling techniques.

## Supplementary Material

Supporting Information

## Figures and Tables

**Figuer 4:**
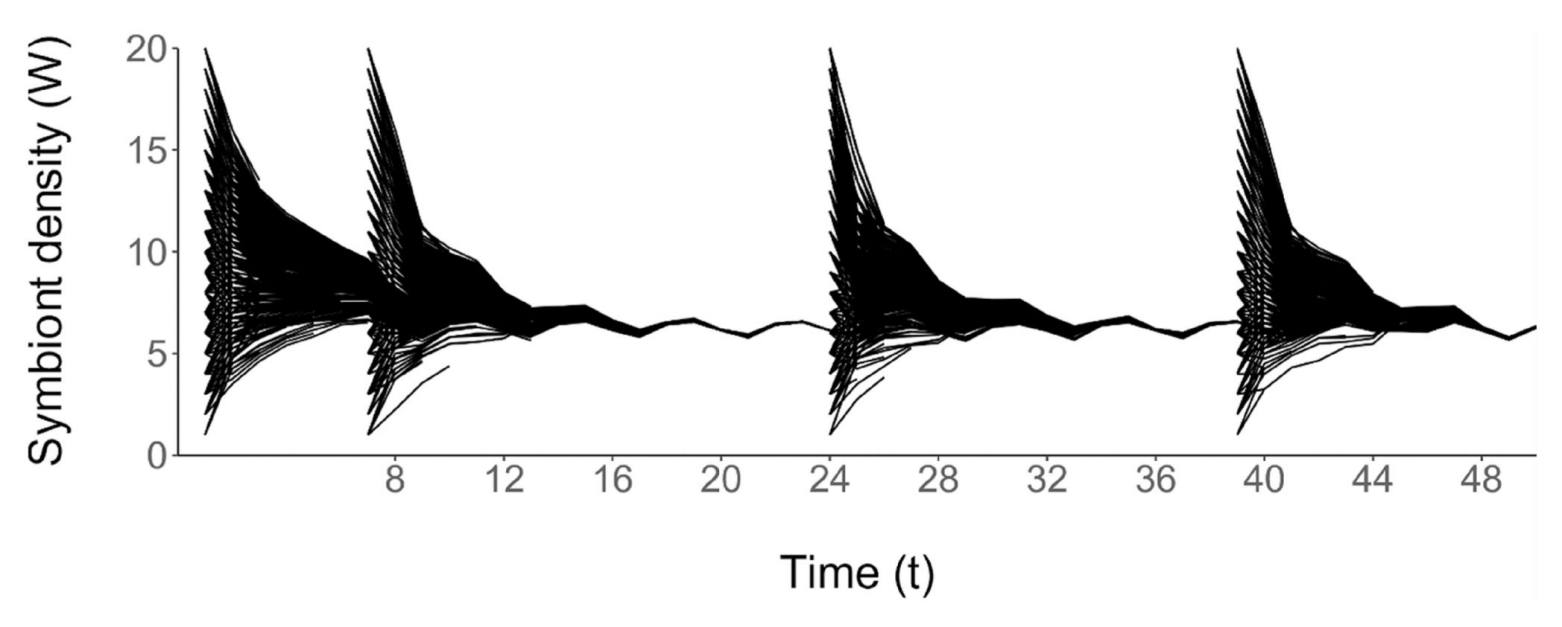
Symbiont density subjected to the optimal strategy of host regulation (ten hosts per state at *t* = 1, 4000 total). Energy reserves and symbiont density are perturbed from the stable state to their starting values at *t* = 7, 24 and 39. Each line indicates the symbiont density of one simulated host. The time steps of reproductive events are indicated on the x-axis.

**Table 1:** Parameters of the base-case model and ranges explored in the sensitivity analysis.

Parameter	Meaning	Value in base-case model	Range explored in sensitivity analysis
*T*	Time horizon, maximum number of time steps hosts can live	50	40–60
*E*_max_	Maximum level of host energy reserves (*E*)	20	–
*W*_max_	Maximum level of symbiont density (*W*)	20	–
*E*_crit_	Minimum level of energy reserves required to reproduce	6	1–14
*W*_crit_	Minimum level of symbiont density required to reproduce	6	1–12
*N*	Energy acquired through feeding, per time step	8	6–15
*μ*	Increase of metabolic expenditure with energy reserves	0.2	0–2
*ω*	Minimum loss from energy reserves per time step (metabolic expenditure)	2	0–5
*α*	Maintenance cost of supporting symbiont population (energy per unit of symbiont density)	0.5	0.1–1.5
*β*	Increase in symbiont population (symbiont density per unit of energy invested)	0.5	0.01–5
*λ*	Proportion of energy reserves transferred to offspring/invested in reproduction	0.3	0.1–0.9
*γ*	Proportion of symbiont transferred to offspring	0.2	0.1–0.9

**Table 2:** Correlation between parameters and the level around which the symbiont density plateaus.

Parameter	*R*	*p*-Value
*T*	0.042	0.400
*N*	**0.360**	**<0.05**
*E*_crit_	–0.060	0.230
*W*_crit_	0.044	0.380
*μ*	**–0.240**	**<0.05**
*ω*	**–0.130**	**<0.05**
*α*	**–0.490**	**<0.05**
*β*	**0.360**	**<0.05**
*λ*	0.031	0.540
*γ*	0.071	0.160

*Note*: Significant relationships at the 5% level are indicated in bold.

**Table 3:** Parameters and functions modified from the base-case model for different host-symbiont ecologies.

Model description	Changes from base-case model
Optimal for symbiont	*B*(*E*(*t*), *W*(*t*); *u*) = *W*_*rep*_
Non-essential symbiont	*W*_crit_ = 0 *S*(*E*(*t*), *W*(*t*); *u*) = (1 − *e*^−^^0.3*E‴*^^(*t*)^) (1 − *e*^−^^0.3(*w*″(*t*)+2)^) *B*(*E*(*t*), *W*(*t*); *u*) = 0.5 *E*_rep_ *W*_rep_ + 5
Symbiont is no longer required by the host for survival after maturation	$S(E(t),W(t);u)=\{\begin{array}{c} \left(1-e^{-0.3E^{\prime \prime \prime }(t)}\right)\left(1-e^{-0.3W^{\prime \prime }(t)}\right),t<8\\ 1-e^{-0.3E^{\prime \prime \prime }(t)},t\geq 8 \end{array}$
A host with a prolonged post-reproductive life stage	The final reproductive event occurs at *t* = 32

**Table 4:** Pairwise interactions of the different strategies of symbiont density regulation for host and symbiont fitness, as determined by pairwise Wilcoxon Rank Sum tests.

	Imperfect	Fixed	Optimized to symbiont	Proportional
Host				
Base-case	0.746	**<0.05**	0.746	**<0.05**
Imperfect	-	**<0.05**	0.746	**<0.05**
Fixed	-	-	0.093	**<0.05**
Optimized to symbiont	-	-	-	**<0.05**
Symbiont				
Base-case	0.177	**<0.05**	0.177	**<0.05**
Imperfect	-	**<0.05**	0.085	**<0.05**
Fixed	-	-	**<0.05**	**<0.05**
Optimized to symbiont	-	-	-	**<0.05**

*Note*: Significant relationships at the 5% level are indicated in bold.

## Data Availability

Code used for analyses is available in Dryad Digital Repository, at https://doi.org/10.5061/dryad.xgxd254nh.
