# Supplementary material for: A theoretical model for host-controlled regulation of symbiont density
Source: J Evol Biol. Author manuscript; Available in PMC 2025 Feb 22. (PMC7617405; doi:10.1111/jeb.14246)
Supplement: Supporting Information [file EMS202803-supplement-Supporting_Information.docx]

*Supplementary Material*

In the base-case model, reproduction begins at t = 8 and occurs every four time steps for the rest of host development. To determine if this schedule for reproduction qualitatively influences the model output we tested alternatives (table S1). The general trend of symbiont density dynamics was consistent with the base-case model.

Table S1. Different reproductive schedules tested for any influence on symbiont density dynamics.

| Time of first reproductive event (t) | Time steps between reproductions | Simulated symbiont density dynamics |
| --- | --- | --- |
| 4 | 4 | Fig S1 |
| 8 | 4 | Base-case (fig 3, main text) |
| 16 | 4 | Fig S2 |
| 16 | 8 | Fig S3 |


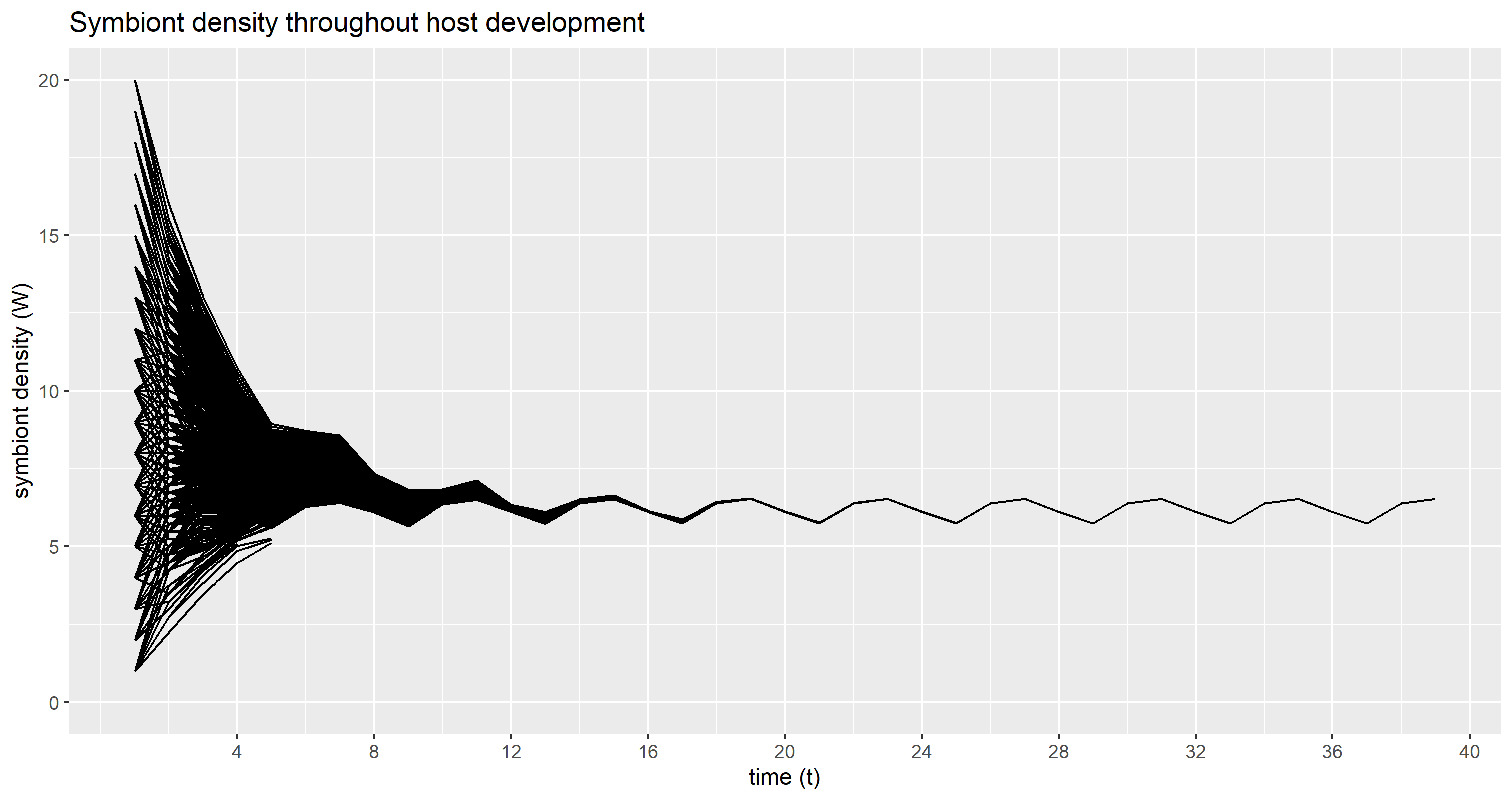


Figure S1. Symbiont density throughout host development where reproductive events occur from t = 4 and every four time steps thereafter.

_
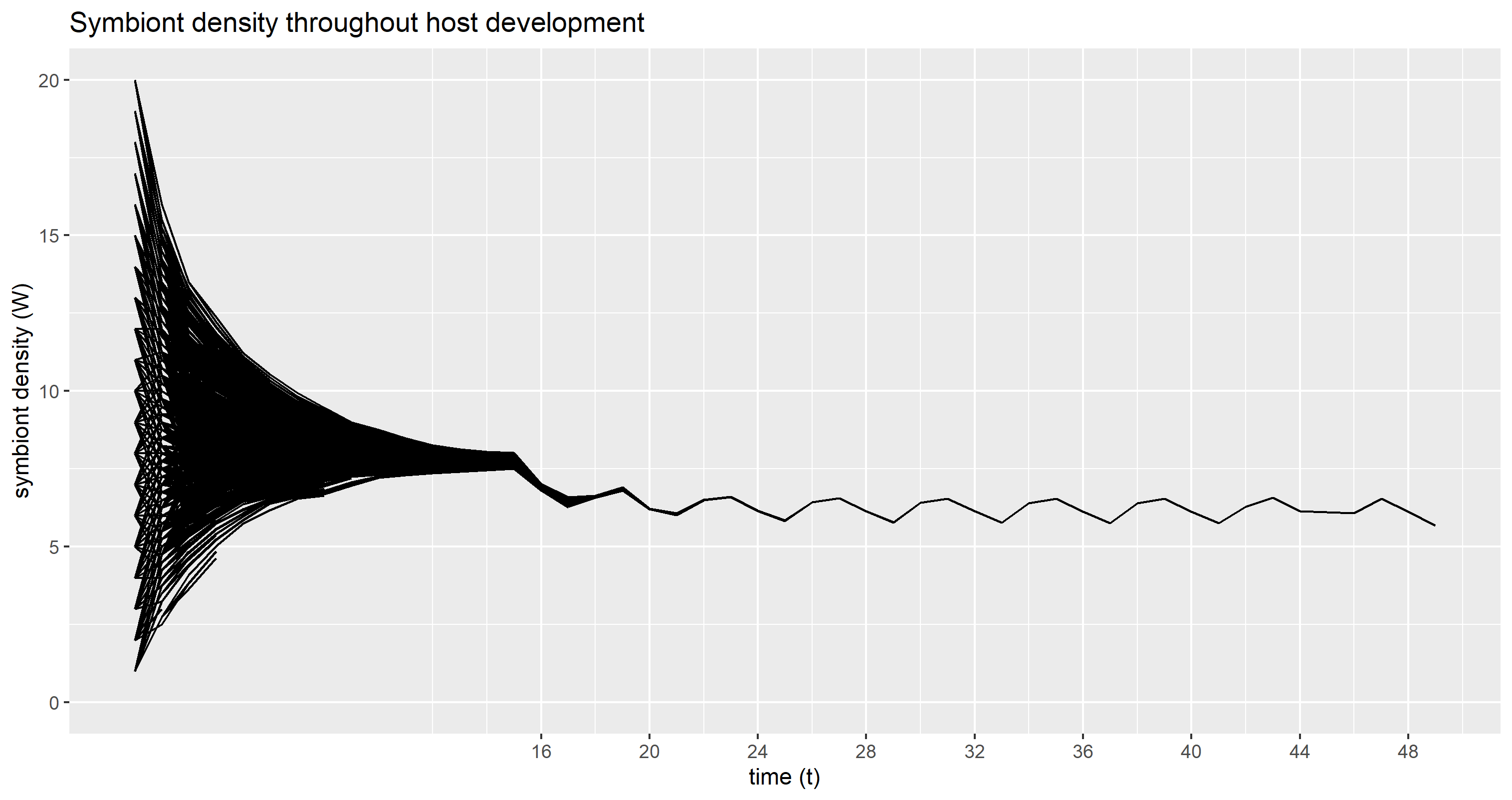
_

Figure S2. Symbiont density throughout host development where reproductive events occur from t = 16 and every four time steps thereafter.


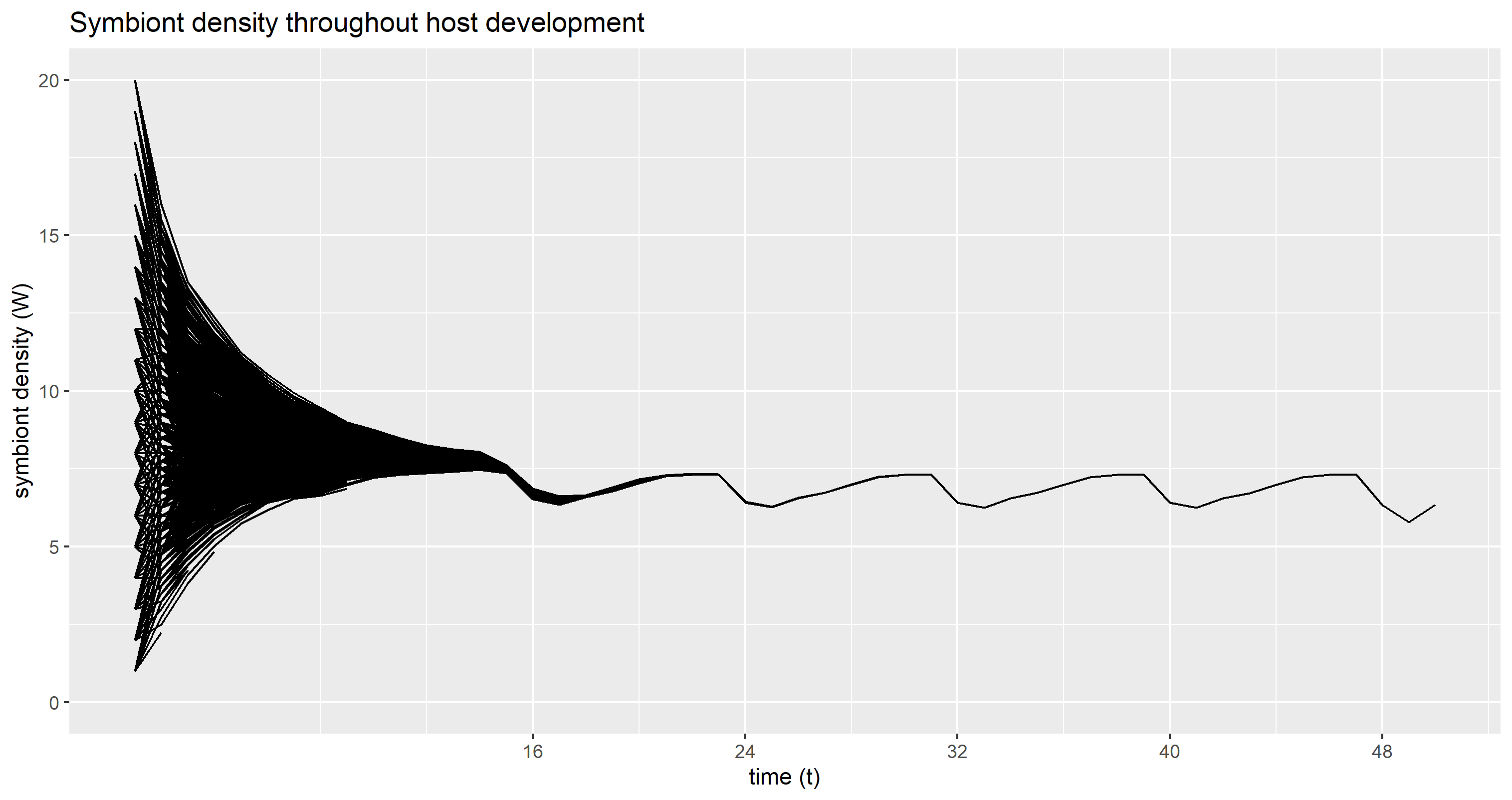


Figure S3. Symbiont density throughout host development where reproductive events occur from t = 8 and every eight time steps thereafter.

The effect of stochasticity in the amount of dietary energy received by hosts was tested, where at each time step N was sampled from the interval [0, 12], with the probability ($p$) of each discrete value being chosen given by a Beta distribution $p\sim Beta(6,3)$. Figure S4 shows the optimal decisions to be made by hosts in each state, given this unpredictability in feeding, and figure S5 the resulting dynamics of symbiont density throughout host development. The strategy for energy allocation is very similar to the base case (fig 2 of main text). Stochasticity introduces noise into the simulated symbiont density dynamics, however the trend of convergence onto one level of symbiont density (≈ 6) is observed (see fig 3 of main text for base case).


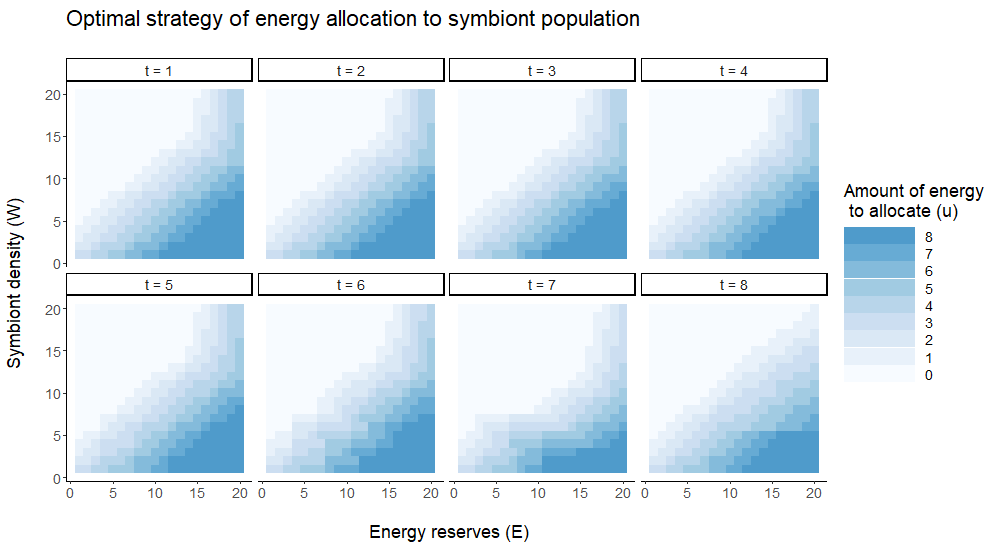


Figure S4. The optimal amount of energy to be allocated to the symbiont population for hosts in each state, for hosts which experience stochasticity in the amount of energy they receive through feeding.


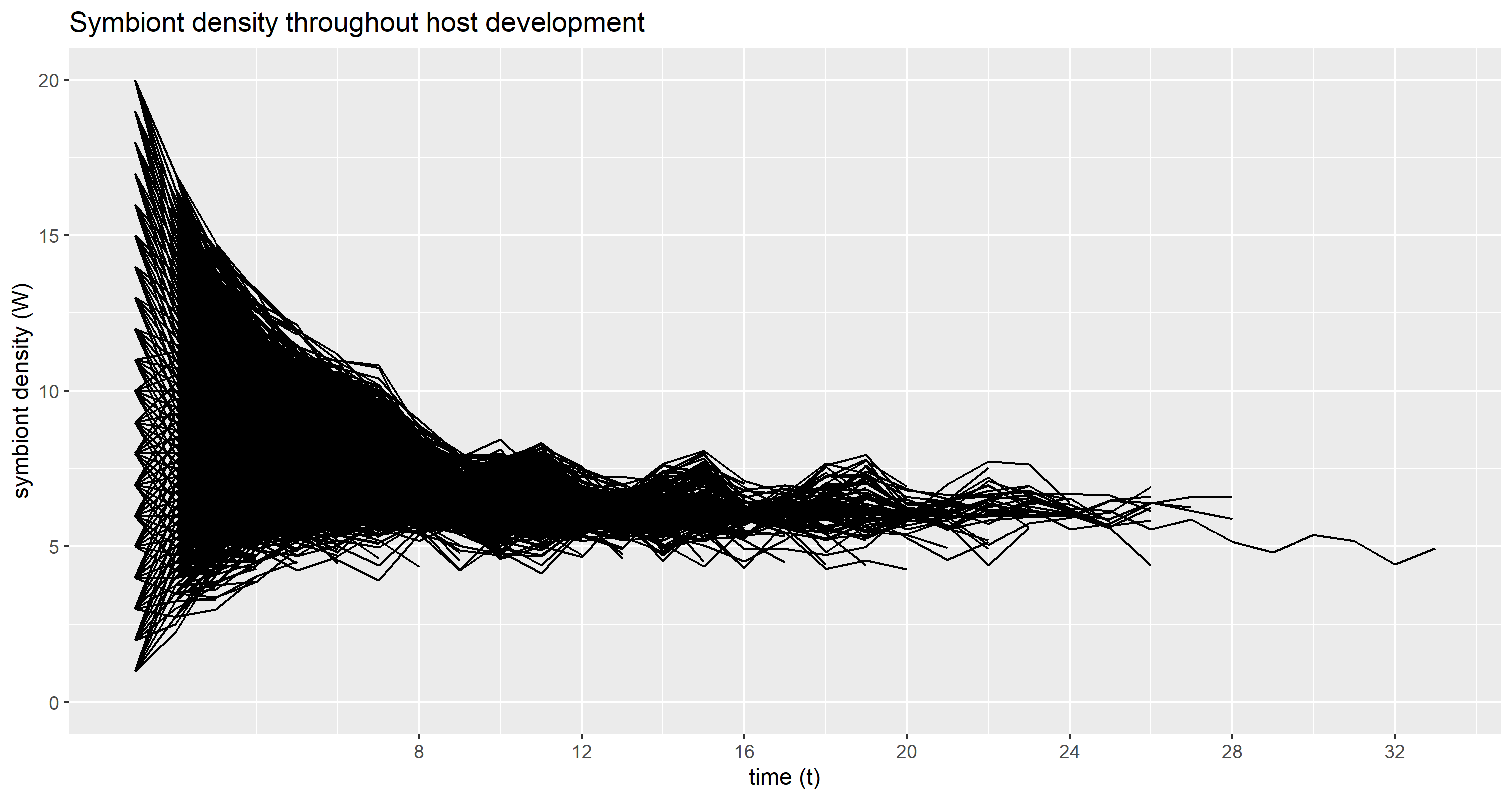


Figure S5. Symbiont density throughout host development for hosts which experience stochasticity in the amount of energy they receive through feeding.

As the dependence of fitness on the energy and symbiont density spent on reproduction is unknown, several functional forms were considered: linear, quadratic and asymptotic (fig. S6). Models were built using each combination of these (table S2) to calculate $B\left( E(t), W(t);u \right)$, and simulations produced to identify any major differences (fig. S7 – S14). The general trend was consistent with the base-case model.

| Relationship between energy reserves spent during reproduction and the immediate contribution to fitness | Relationship between symbiont density transmitted to offspring and the immediate contribution to fitness | Equation | Simulated symbiont density dynamics |
| --- | --- | --- | --- |
| linear | linear | $E_{rep} W_{rep}$ | Base-case (Fig 3, main text) |
| linear | quadratic | $E_{rep} (4-0.25\left( W_{rep}-4 \right)^{2})$ | Fig. S7 |
| linear | asymptotic | $E_{rep} (4\left( 1-\exp\left( -W_{rep} \right) \right))$ | Fig. S8 |
| quadratic | linear | $(4-0.25{(E_{rep}-4)}^{2} {) W}_{rep}$ | Fig. S9 |
| quadratic | quadratic | $\left( 4-0.25\left( E_{rep}-4 \right)^{2} \right)(4-0.25\left( W_{rep}-4 \right)^{2})$ | Fig. S10 |
| quadratic | asymptotic | $\left( 4-0.25\left( E_{rep}-4 \right)^{2} \right)(4\left( 1-\exp\left( -W_{rep} \right) \right))$ | Fig. S11 |
| asymptotic | linear | $(4\left( 1-\exp\left( -E_{rep} \right) \right))W_{rep}$ | Fig. S12 |
| asymptotic | quadratic | $\left( 4\left( 1-\exp\left( -E_{rep} \right) \right) \right)(4-0.25\left( W_{rep}-4 \right)^{2})$ | Fig. S13 |
| asymptotic | asymptotic | $\left( 4\left( 1-\exp\left( -E_{rep} \right) \right) \right)(4\left( 1-\exp\left( -W_{rep} \right) \right))$ | Fig S14 |

Table S2. Different functional forms considered for the dependence of fitness on host energy reserves and symbiont density.


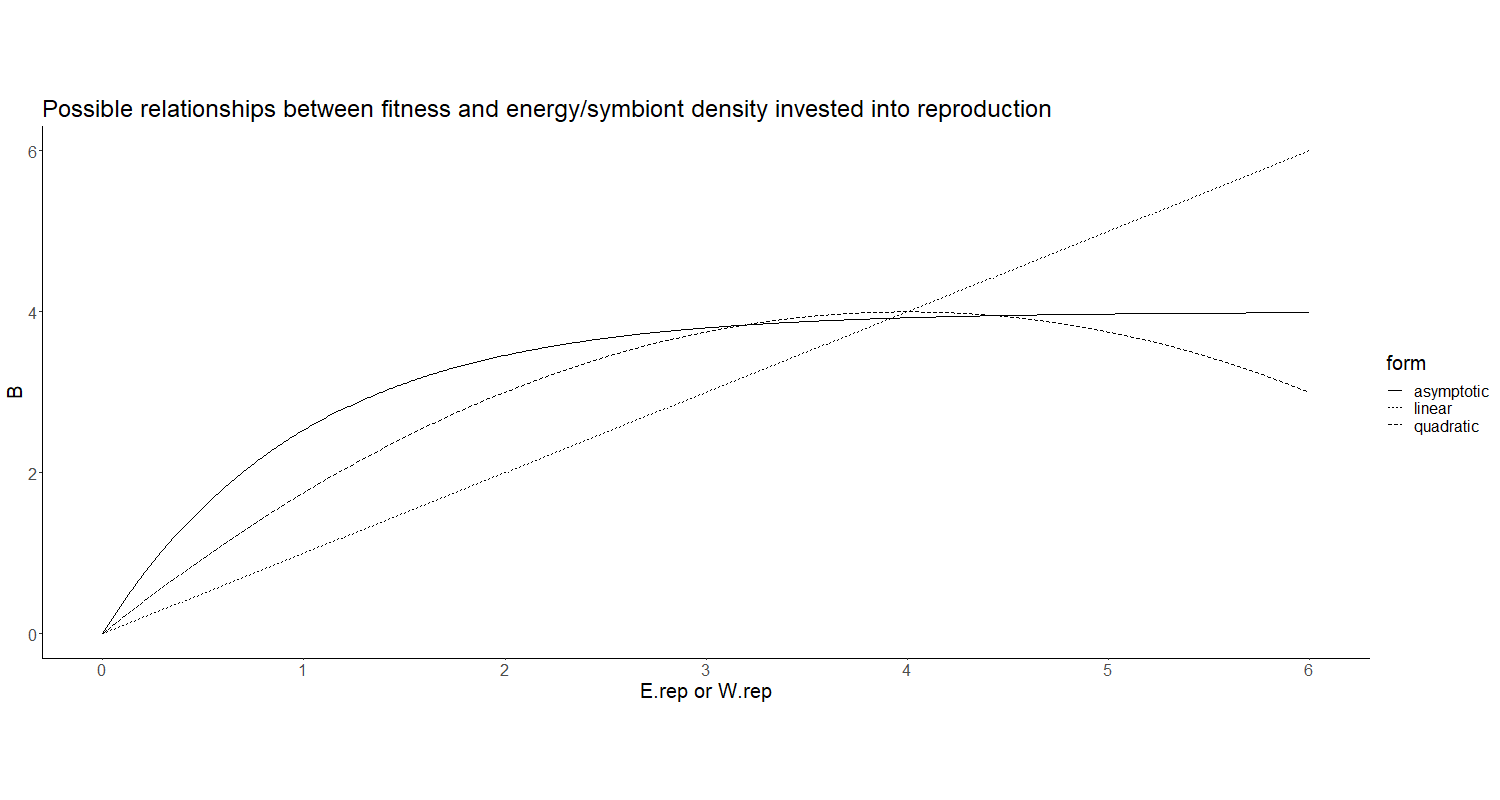


Figure S6. The possible forms for the relationship between the energy reserves invested into reproduction/ the symbiont density transmitted to offspring and the immediate contribution to fitness (B).


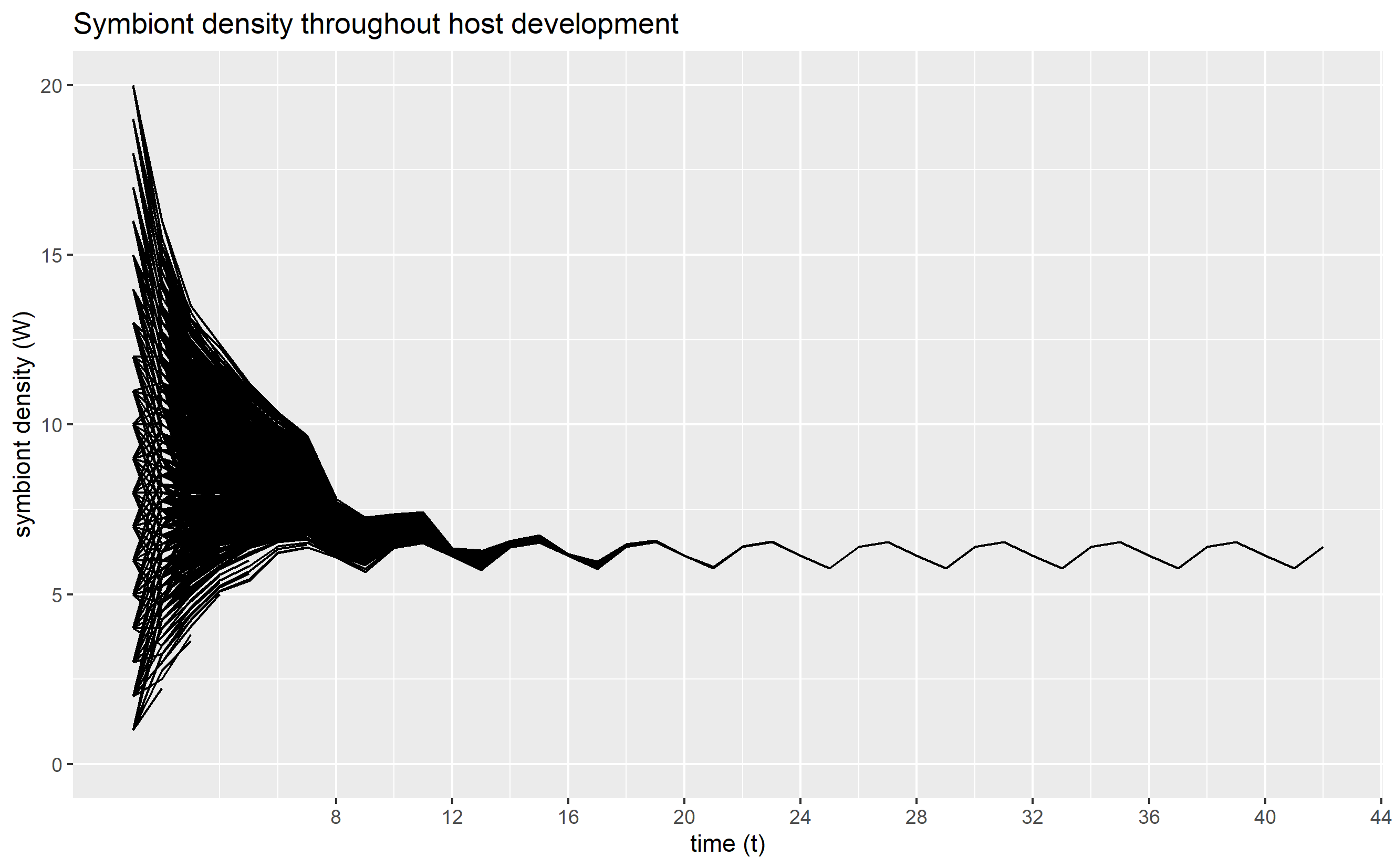


Figure S7. Symbiont density throughout host development where B is linearly dependent on E_rep_ and quadratically dependent on W_rep_

_
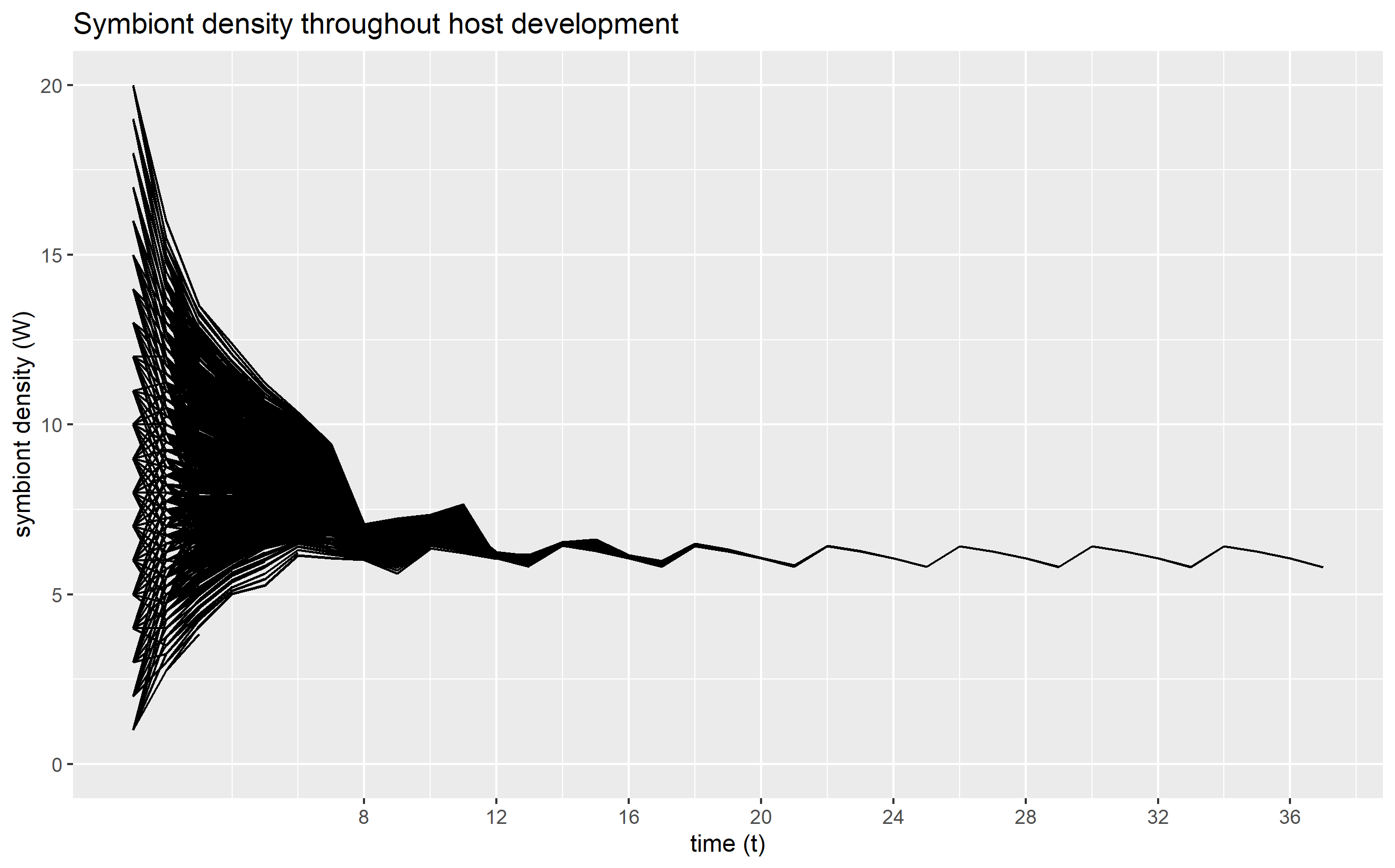
_

Figure S8. Symbiont density throughout host development where B is linearly dependent on E_rep_ and asymptotically dependent on W_rep_

_
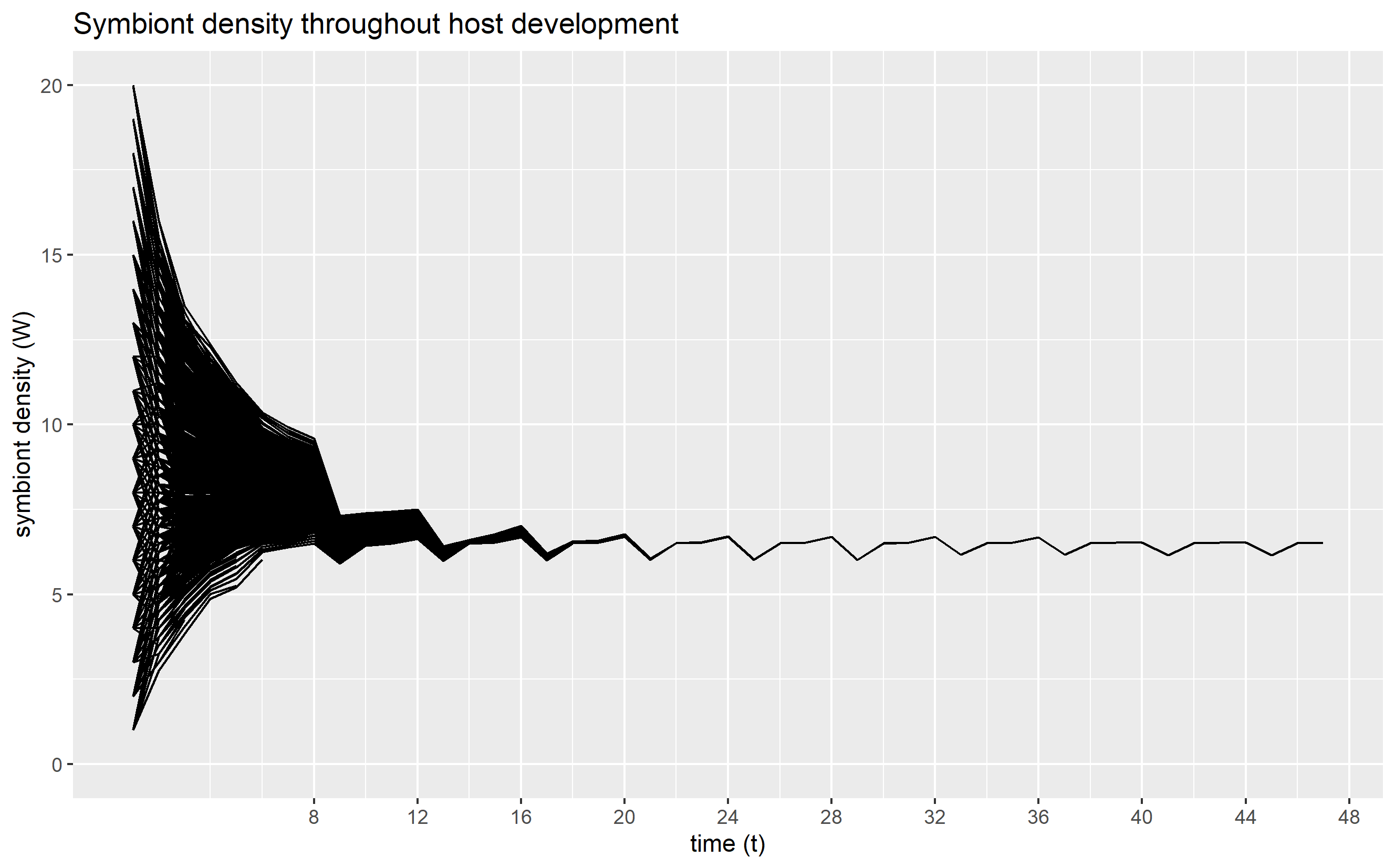
_

Figure S9. Symbiont density throughout host development where B is quadratically dependent on E_rep_ and linearly dependent on W_rep_

_
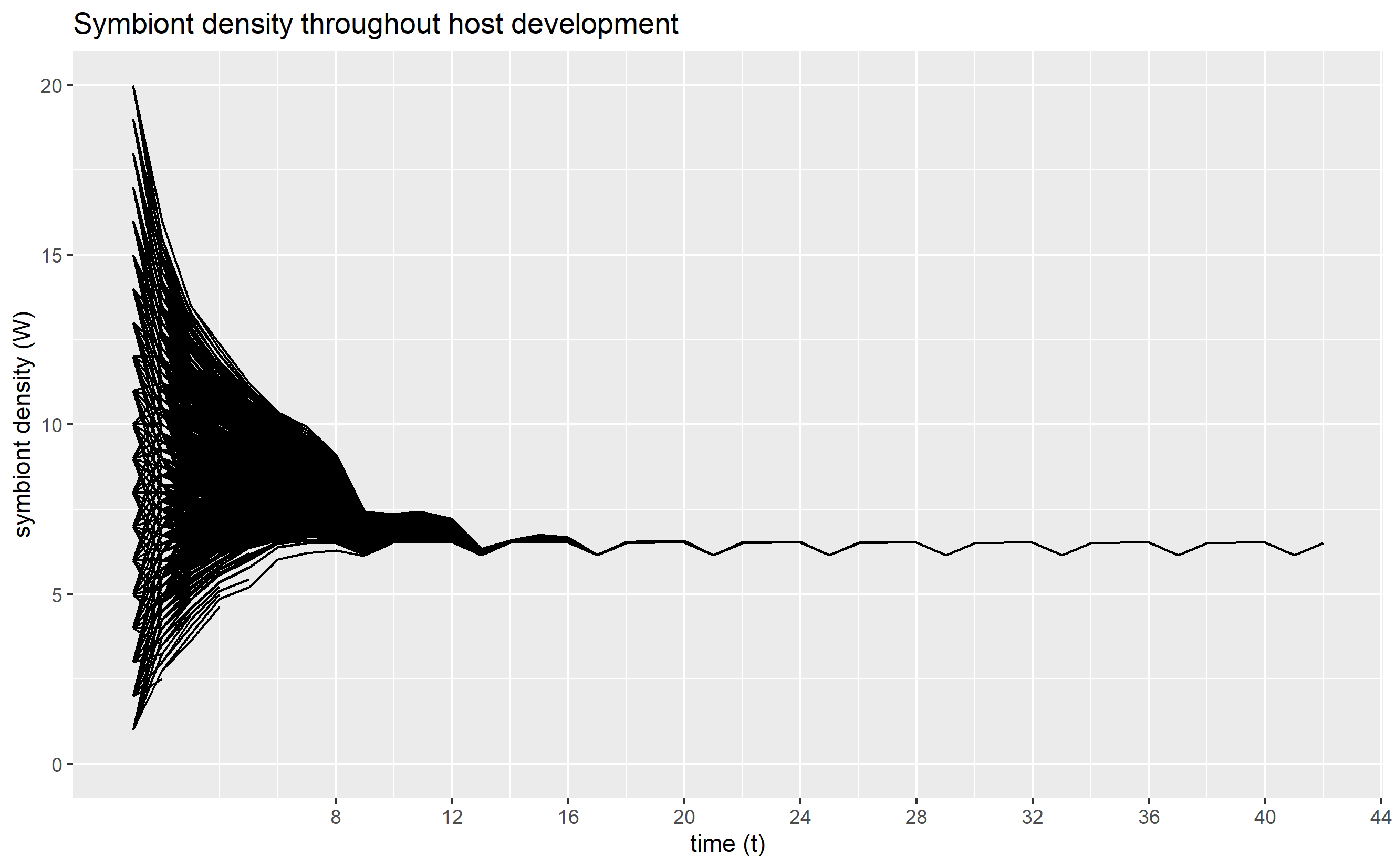
_

Figure S10. Symbiont density throughout host development where B is quadratically dependent on E_rep_ and W_rep_

_
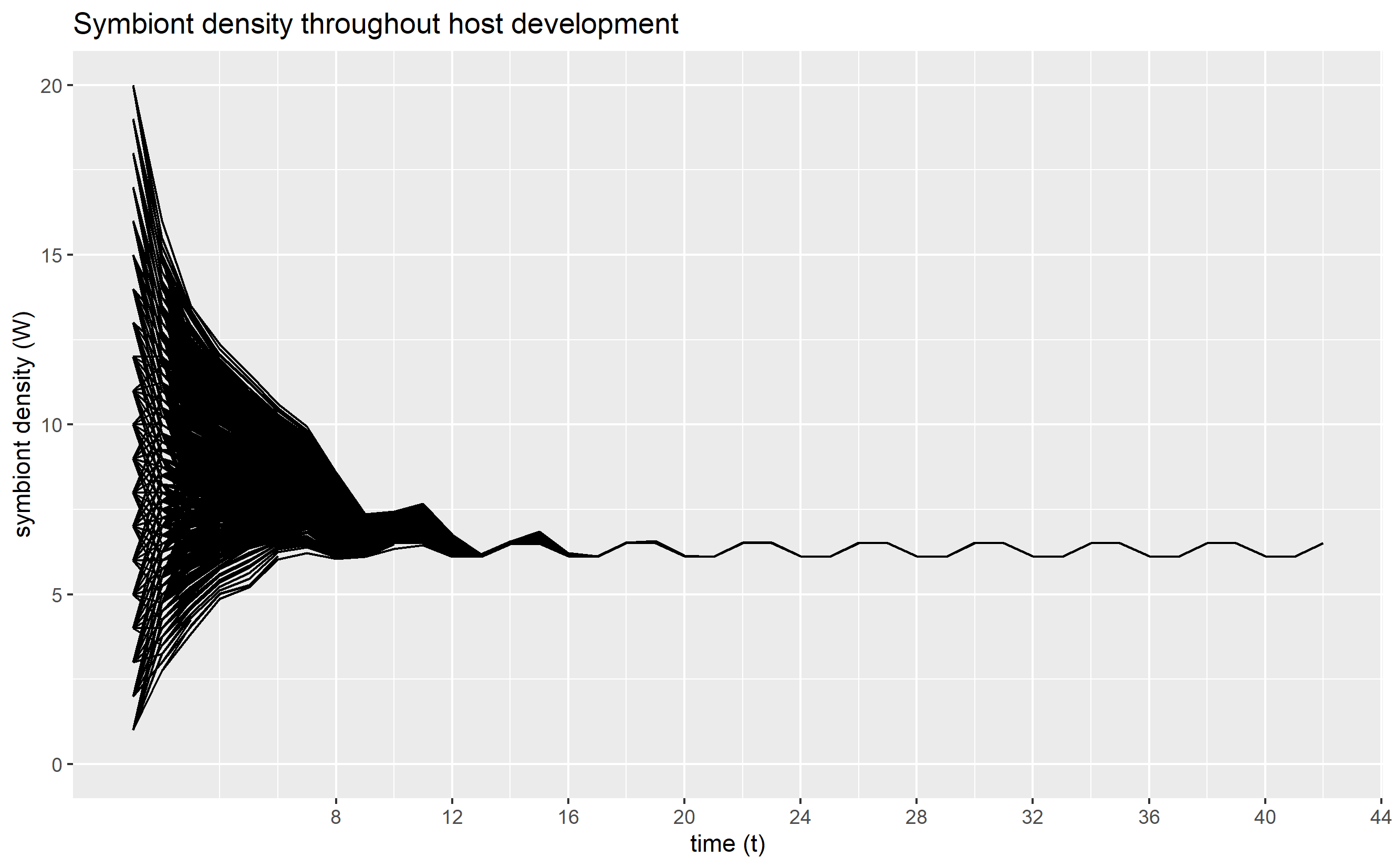
_

Figure S11. Symbiont density throughout host development where B is quadratically dependent on E_rep_ and asymptotically dependent on W_rep_

_
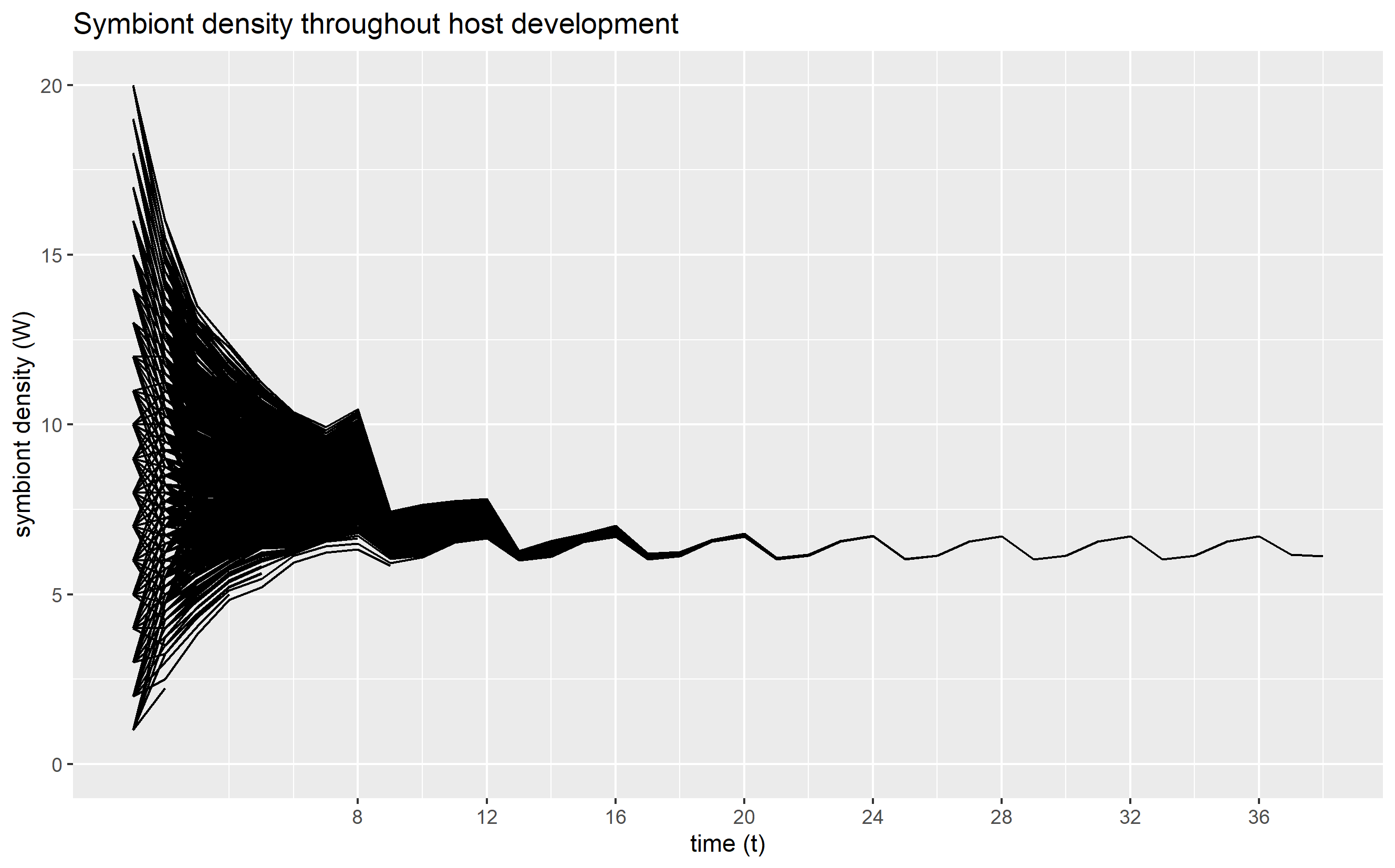
_

Figure S12. Symbiont density throughout host development where B is asymptotically dependent on E_rep_ and linearly dependent on W_rep_

_
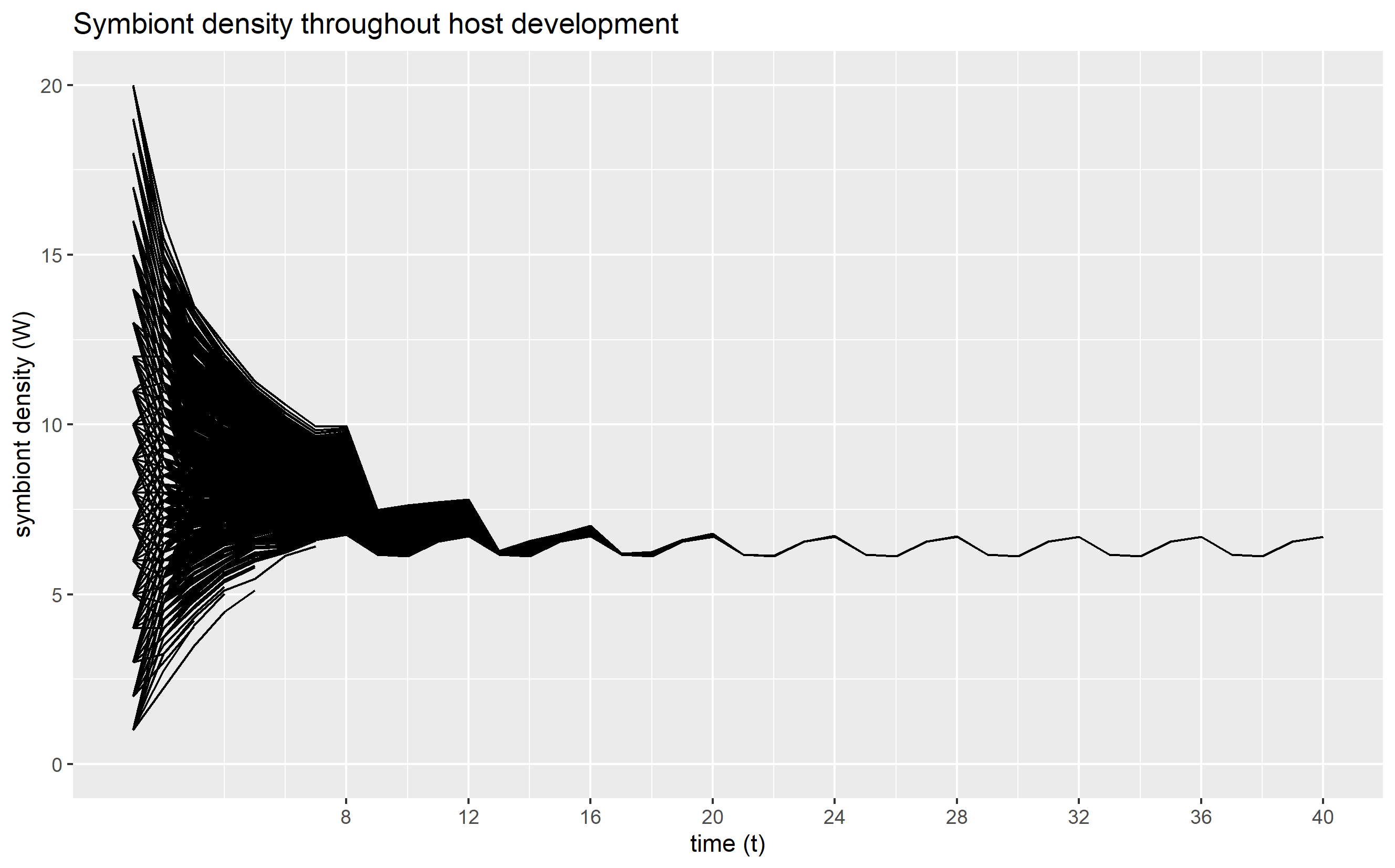
_

Figure S13. Symbiont density throughout host development where B is asymptotically dependent on E_rep_ and quadratically dependent on W_rep._

_
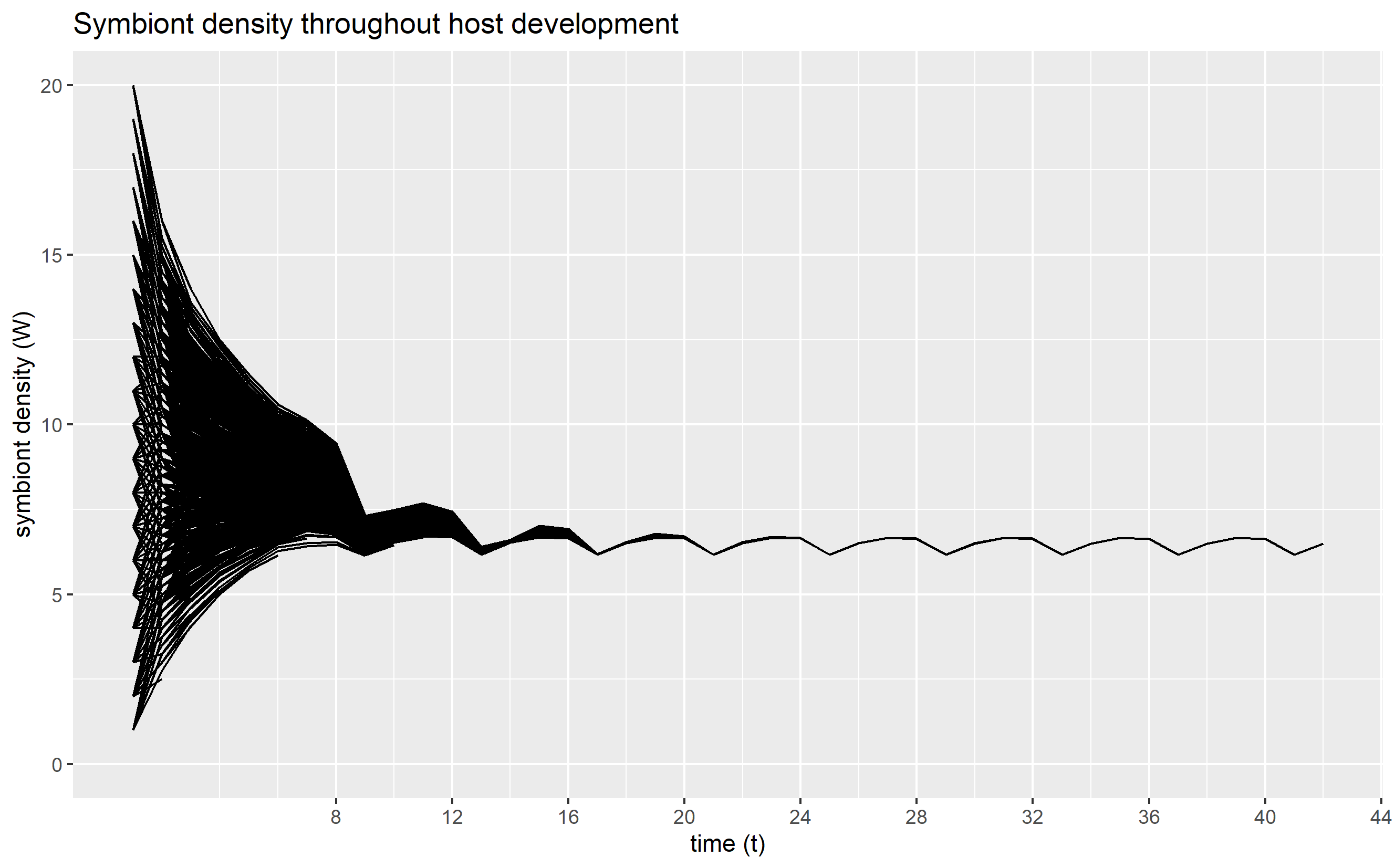
_

Figure S14. Symbiont density throughout host development where B is asymptotically dependent on E_rep_ and W_rep_
